# A Co-essentiality Network of Cancer Driver Genes Better Prioritizes Anticancer Drugs

**DOI:** 10.1093/gpbjnl/qzaf070

**Published:** 2025-09-26

**Authors:** Kwanghwan Lee, Donghyo Kim, Inhae Kim, Juhee Lee, Doyeon Ha, Seongsu Lim, Eunjee Kim, Sin-Hyeog Im, Kunyoo Shin, Sanguk Kim

**Affiliations:** Department of Life Sciences, Pohang University of Science and Technology, Pohang 790-784, Republic of Korea; Department of Life Sciences, Pohang University of Science and Technology, Pohang 790-784, Republic of Korea; ImmunoBiome Inc., Bio Open Innovation Center, Pohang 790-784, Republic of Korea; Institute of Molecular Biology and Genetics, Seoul National University, Seoul 08826, Republic of Korea; School of Biological Sciences, College of Natural Sciences, Seoul National University, Seoul 08826, Republic of Korea; Department of Life Sciences, Pohang University of Science and Technology, Pohang 790-784, Republic of Korea; School of Interdisciplinary Bioscience and Bioengineering, Pohang University of Science and Technology, Pohang 790-784, Republic of Korea; Institute of Molecular Biology and Genetics, Seoul National University, Seoul 08826, Republic of Korea; School of Biological Sciences, College of Natural Sciences, Seoul National University, Seoul 08826, Republic of Korea; Department of Life Sciences, Pohang University of Science and Technology, Pohang 790-784, Republic of Korea; ImmunoBiome Inc., Bio Open Innovation Center, Pohang 790-784, Republic of Korea; Institute of Molecular Biology and Genetics, Seoul National University, Seoul 08826, Republic of Korea; School of Biological Sciences, College of Natural Sciences, Seoul National University, Seoul 08826, Republic of Korea; Department of Life Sciences, Pohang University of Science and Technology, Pohang 790-784, Republic of Korea

**Keywords:** Anticancer therapy, Network medicine, Disease module, CRISPR screening, Precision oncology

## Abstract

Diverse molecular networks have been extensively studied to discover therapeutic targets and repurpose approved drugs. However, it is necessary to select a suitable network since the performance of network medicine relies heavily on the completeness and characteristics of the selected network. Although a network using gene essentiality in cancer cells could be an effective platform for identifying anticancer targets, efforts to apply these networks to therapeutic applications have been limited. We constructed a phenotype-level network using co-essentiality relationships among genes from CRISPR screens across 769 cancer cell lines to discover therapeutic targets for diverse cancer types. By leveraging cancer driver genes and network propagation, we found that the co-essentiality network better prioritized anticancer targets and biomarkers and predicted more precise drug responses in cancer cells than other molecular networks. The co-essentiality network outperformed conventional molecular networks in drug repurposing and was validated *in silico* by clinical trial records. Notably, the co-essentiality network identified 30 repurposed drugs that the other networks have not yet covered, and we showcased three approved drugs repurposed for lung adenocarcinoma (atovaquone, eflornithine, and teriflunomide). Our study provides a novel network for precision oncology to improve the identification of therapeutic targets in specific cancers.

## Graphical abstract

**Figure:**
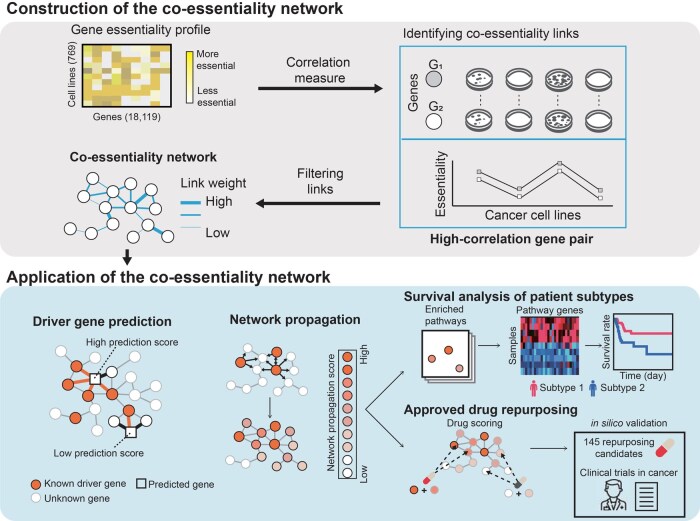

## Introduction

Over the past few years, various anticancer drugs have been developed for diverse cancer types [1]. However, the overall clinical efficacy of approved drugs remains limited. Thus, identifying targetable alterations is urgently needed for the success of anticancer therapy. To achieve precision oncology, diverse tasks must be performed, such as identifying driver genes and discovering drug targets for specific cancer types.

Network-based approaches support precision oncology to identify robust anticancer targets or biomarkers linked to known disease genes [2,3], since genes related to disease phenotypes cooperate and cluster in the network [4]. We recently identified biomarkers of chemotherapy and immunotherapy by propagating the relatedness of therapeutic agents from drug targets to their neighbors in a protein–protein interaction (PPI) network [5,6]. Cheng et al. developed an *in silico* cancer drug repurposing framework using network modules derived from gene co-expression and PPI networks [7].

However, it remains to be seen which network is suitable for precision oncology, even though the choice of network is crucial to the performance of network-based approaches. It has been shown that network topology is a critical factor in improving the identification of disease genes. Huang et al. showed that the performance gap could exceed 5000× between the networks, from the highest to the lowest [8]. Buphamalai et al. also reported that each network is relevant to specific tasks [9]. Therefore, rather than relying on a one-size-fits-all network, we designed and evaluated networks that perform best on the tasks.

Co-essentiality networks, composed of genes with similar knockout essentiality profiles across various cancer cell lines, can be useful for identifying therapeutic targets in cancer. While several co-essentiality networks have been used for gene function prediction [10,11], the benefits of using them in drug discovery still need to be assessed. For example, it has been reported that genes with similar essentiality profiles tend to have similar biological functions [10]. Co-essentiality networks have been applied to discover new functions of genes [11–13] and to infer genes into the same functional complexes [14] or metabolic pathways [15]. However, attempts to identify cancer drug targets using co-essentiality networks have been limited to discovering surrogate targets for several challenging target proteins [12], despite the therapeutic opportunities the essentiality phenotype might possess.

The potential of co-essentiality networks for drug repurposing remains particularly promising compared to conventional approaches. Most existing drug repurposing methods have relied on PPI networks [7,16] or gene co-expression networks [17,18], which may have limitations in capturing functional relationships relevant to drug response. A co-essentiality network built from cancer cell line data may offer complementary insights by incorporating cellular fitness phenotypes. These phenotype-level relationships could provide additional perspective on drug effects compared to physical interactions or expression correlations. To our knowledge, this study represents the first comprehensive evaluation of co-essentiality networks for drug repurposing applications.

In this study, we aimed to assess diverse *in silico* frameworks using a co-essentiality network to identify novel anticancer drugs for specific cancer types and investigate the advantages of this network compared with conventional molecular networks. We found that the co-essentiality network was able to prioritize cancer-type-specific therapeutic targets and discover drug repurposing candidates. The co-essentiality links formed highly clustered network modules of potential therapeutic targets. Moreover, the co-essentiality network predicted more precise drug responses in cancer cells than other molecular networks. We anticipate that the co-essentiality network will be a valuable resource for precision oncology and provide new therapeutic opportunities for cancer patients.

## Results

### Construction of the co-essentiality network for drug discovery in cancer

**Figure 1:**
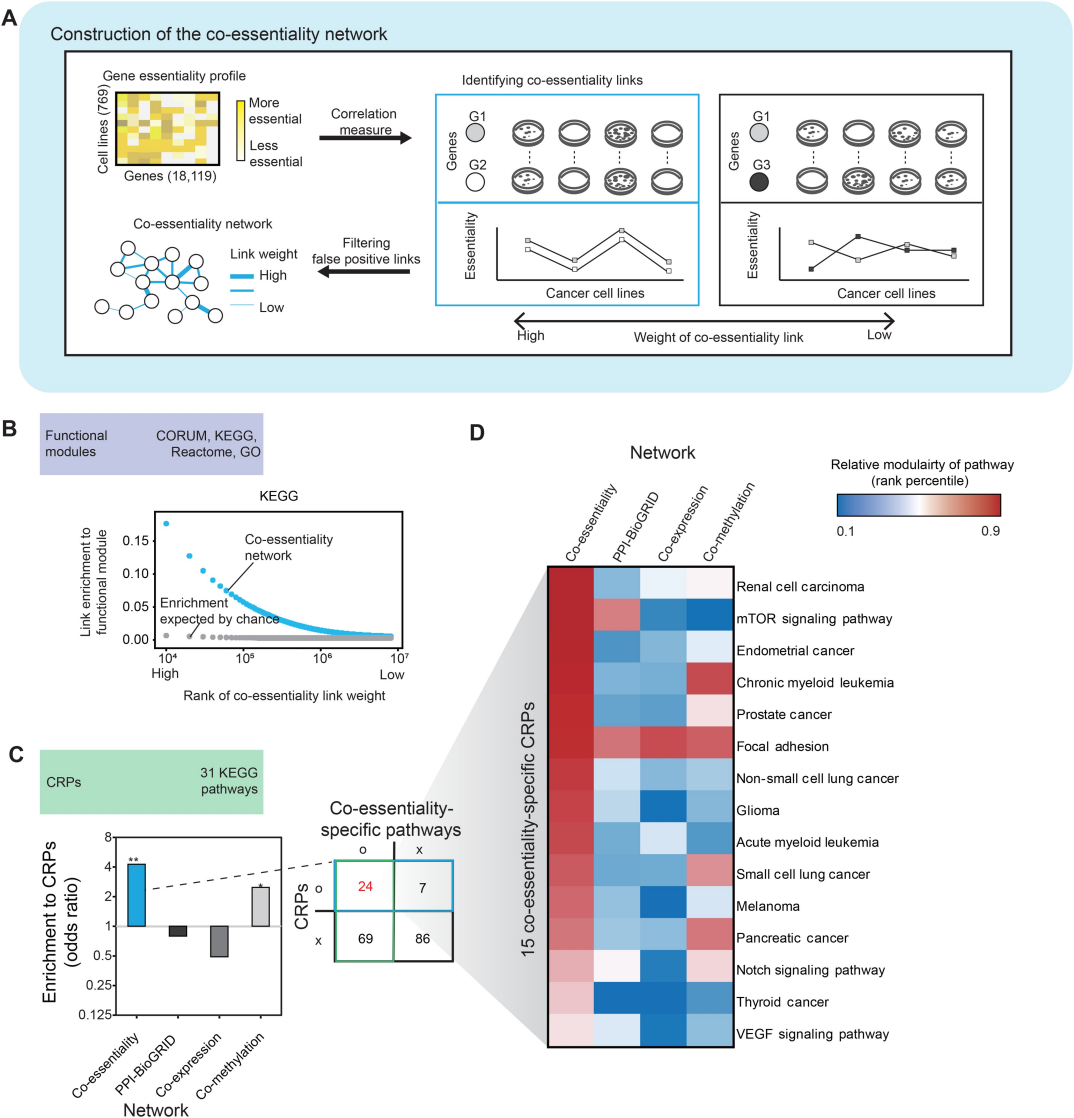
Construction of the co-essentiality network and validation for cancer association **A**. Schematic illustration of constructing the co-essentiality network and its validation. **B**. Enrichment of the co-essentiality links to KEGG pathways. Co-essentiality links were ranked and binned (*n* = 10,000). Enrichment of the co-essentiality links in each bin is shown using blue dots. The expected enrichment of co-essentiality links in each bin is shown using gray dots. **C**. Enrichment of network links to 31 KEGG CRPs for four networks: the co-essentiality network, PPI network (BioGRID), co-expression network, and co-methylation network (left). The contingency table of KEGG pathways was created based on two criteria: inclusion in CRPs and inclusion in co-essentiality-specific pathways (right). “O” and “X” indicate whether a pathway was included in each category. The significance of pathway enrichment was measured by Fisher’s exact test (*, *P* < 0.05; **, *P* < 0.01). **D**. The relative modularity of 15 pathways overlapped between the 31 CRPs and the 93 co-essentiality-specific KEGG pathways. CRP, cancer-related pathway; PPI, protein–protein interaction.

To construct a network for the identification of therapeutic targets and drug repurposing candidates for each cancer type, co-essentiality links were inferred from the correlations in the gene essentiality profiles of cancer cells (**Figure 1**A). A co-essentiality network was constructed with 18,119 genes and 8,105,180 co-essentiality links based on gene-level essentiality scores using a CRISPR screening dataset from the Cancer Dependency Map (DepMap) project [19]. Co-essentiality links represent the similarity of essentiality profiles between two genes across cancer cells and are measured using the context likelihood of relatedness (CLR) algorithm [20]. To validate that the co-essentiality links from our method are relevant to biological function, we tested whether these links are enriched in curated pathways. We found that the co-essentiality links indicated a strong functional relationship between the two genes (Figure 1B, Figure S1), resulting in similar growth phenotypes across various cancer cell lines. For example, in Kyoto Encyclopedia of Genes and Genomes (KEGG) pathways [21], gene pairs with greater link weights were likely to be within the same pathways (Figure 1B, blue dots), and this tendency was higher than expected by chance (Figure 1B, gray dots). Similarly, gene pairs with greater link weights likely resided within the same biological modules from five independent datasets: protein complexes from the CORUM database [22], molecular pathways from Reactome, a curated pathway database [23], and Gene Ontology (GO) annotations [biological process (BP), molecular function (MF), and cellular component (CC )] [24].

### Effectiveness of the co-essentiality network in identifying cancer-related pathways

We found that the co-essentiality network depicts cancer-related pathways (CRPs) related to oncogenesis and hallmarks of cancer. To investigate the advantages of using the co-essentiality network, we compared it with three other molecular networks: a PPI network (BioGRID; 18,708 nodes; 434,527 links) [25], a co-expression network (19,120 nodes; 12,759,793 links), and a co-methylation network (16,333 nodes; 10,193,089 links).

The co-essentiality network showed a higher enrichment of CRPs (Figure 1C, left panel) with an odds ratio of 4.27, compared with the PPI network (odds ratio = 0.79), the co-expression network (odds ratio = 0.49), and the co-methylation network (odds ratio = 2.49). Co-essentiality links showed significant enrichment to CRPs (Fisher’s exact test, *P* = 1.34 × 10^−3^), whereas PPI and co-expression links did not show significant enrichment to CRPs. Among 31 CRPs, 24 had high relative modularity in the co-essentiality network (co-essentiality specific pathways) and were greater than 15.5 CRPs, which was expected by chance (Figure 1C right panel; Table S1).

Using the median relative modularity value as a threshold for each network, we found that the co-essentiality network showed significant enrichment of CRPs (Figure 1C, left panel) with an odds ratio of 4.27 (Fisher’s exact test, *P* = 1.34 × 10^−3^). In contrast, the PPI network (odds ratio = 0.79, *P* = 0.694) and co-expression network (odds ratio = 0.49, *P* = 0.114) showed depletion of CRPs, although these results were not statistically significant. The co-methylation network showed moderate enrichment (odds ratio = 2.49, *P* = 0.0307). This pattern was further validated by gene set enrichment analysis (GSEA). GSEA confirmed significant enrichment of CRPs at high relative modularity values in the co-essentiality network [normalized enrichment score (NES) = 1.53, false discovery rate (FDR) = 0.007], while other networks showed no significance (Figure S2A). Furthermore, a comparative analysis of relative modularity distributions demonstrated that CRPs consistently exhibited significantly higher modularity in the co-essentiality network (median = 0.710) compared to both the PPI network (median = 0.465, *P* = 7.09 × 10^−3^) and co-expression network (median = 0.414, *P* = 1.72 × 10^−3^). Although CRPs showed comparable modularity to the co-methylation network (median = 0.663, *P* = 0.636; Figure S2B), the co-essentiality network still performed better in capturing CRPs. These findings provide compelling evidence that the co-essentiality network captures the cohesive structural characteristics of CRPs more effectively compared to other molecular interaction networks.

For example, in one of the 24 CRPs, the mTOR signalling pathway (KEGG: hsa04150), which is targeted by many anticancer drugs [26,27], the co-essentiality network showed higher modularity than the other networks (Figure 1D, rank percentile = 0.898, 0.697, 0.177, and 0.109 of relative modularity for the co-essentiality network, BioGRID, co-expression network, and co-methylation network, respectively). To determine whether co-essentiality links were likely to be found among gene pairs in CRPs, we measured the relative modularity of genes in each KEGG pathway and compared it with three other molecular networks (see Materials and methods; Table S1). Since modularity is biased by node degree, we used a degree-controlled modularity measure normalized by a permutation test with degree-matched random nodes. These results suggest that co-essentiality links can detect more oncogenic relationships compared with other molecular links, thereby improving the identification of genes critical for cancer treatment.

### High modularity of cancer driver genes in the co-essentiality network

**Figure 2:**
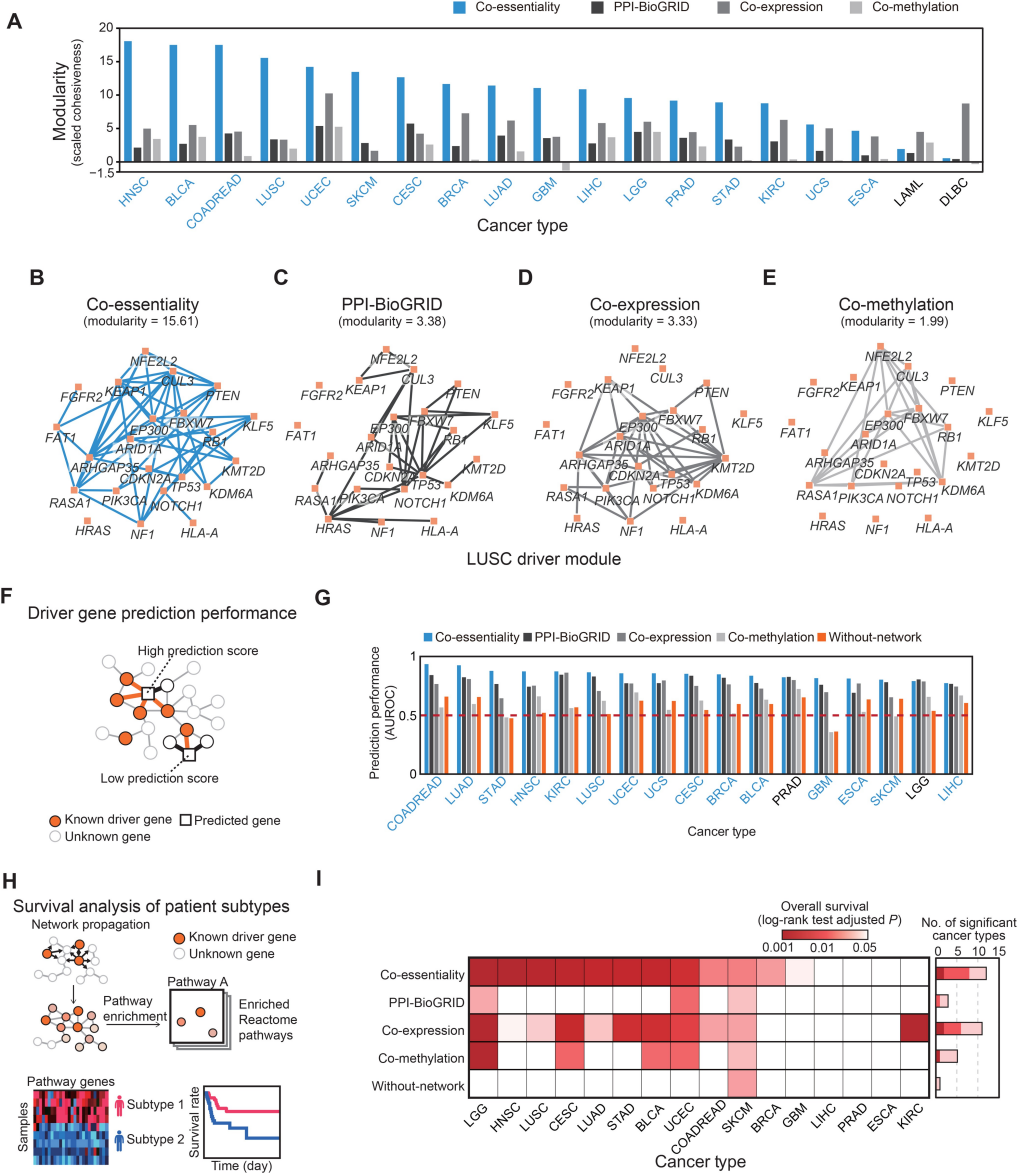
High modularity of cancer driver genes in the co-essentiality network is the key to driver gene identification and patient stratification **A**. Modularity calculated from a subnetwork of driver genes across 19 TCGA cancer types in four networks: co-essentiality, PPI-BioGRID, co-expression, and co-methylation. For blue-colored cancer types, the co-essentiality network showed the highest modularity among the four networks. **B**.–**E**. Illustrations of the subnetwork of LUSC driver genes in four networks: co-essentiality (B), PPI-BioGRID (C), co-expression (D), and co-methylation (E). **F**. A schematic illustration of driver gene identification using the co-essentiality network. **G**. Driver gene identification performance in the four networks (co-essentiality, PPI-BioGRID, co-expression, and co-methylation), as well as the “without-network” control. For blue-colored cancer types, the co-essentiality network showed the best performance among the four networks. **H**. A schematic illustration of TCGA patient stratification using the co-essentiality network. **I**. Results for patient stratification using the four networks and the “without-network” control. A heatmap showing the significance of survival difference between two patient groups stratified by each network tested using the log-rank test. Benjamini-Hochberg method to correct for multiple testing when analyzing *P* values from log-rank test across cancer types. TCGA, The Cancer Genome Atlas; BLCA, bladder urothelial carcinoma; BRCA, breast invasive carcinoma; CESC, cervical squamous cell carcinoma and endocervical adenocarcinoma; COADREAD, colorectal adenocarcinoma; DLBC, diffuse large B-cell lymphoma; ESCA, esophageal carcinoma; GBM, glioblastoma multiforme; HNSC, head and neck squamous cell carcinoma; KIRC, kidney renal clear cell carcinoma; LAML, acute myeloid leukemia; LGG, brain lower grade glioma; LIHC, liver hepatocellular carcinoma; LUAD, lung adenocarcinoma; LUSC, lung squamous cell carcinoma; PRAD, prostate adenocarcinoma; SKCM, skin cutaneous melanoma; STAD, stomach adenocarcinoma; UCEC, uterine corpus endometrial carcinoma; UCS, uterine carcinosarcoma; AUROC, area under the receiver operating characteristic curve.

To verify the effectiveness of co-essentiality links for identifying therapeutic targets in cancer, we investigated the modularity of disease genes in the network, since this is indicative of the relevance of underlying information to a particular disease phenotype [9]. We used cancer driver genes, defined as genes whose mutations can cause cancer, as disease genes. The co-essentiality links resulted in a denser module of cancer driver genes compared with other molecular links. For 17 out of 19 cancer types, the modularity (*m*) of driver genes was the highest in the co-essentiality network compared with the other three molecular networks (**Figure 2**A). Since modularity measures can be affected by node degree, which is high in the co-essentiality network, we used more conservative measures, the degree-controlled modularity (Z-score) measure normalized by the permutation test (see Materials and methods). For instance, in the case of lung squamous cell carcinoma (LUSC), the co-essentiality network showed the highest modularity for driver genes (*m*_co-essentiality network_ = 15.61) among all molecular networks (*m*_PPI-BioGRID_ = 3.38, *m*_co-expression network_ = 3.33, and *m*_co-methylation network_ = 1.99; Figure 2B−E, Figure S3). For example, comparing the driver genes of LUSC (Figure 2B) among the networks (Figure 2C−E), the subnetwork of driver genes in the co-essentiality network was more densely connected. *FAT1* and *FGFR2* were connected to the subnetwork of LUSC driver genes in the co-essentiality network, but were not connected to other molecular networks. *FAT1* showed co-essentiality interactions with *RASA1*, *CUL3*, and *ARHGAP35*. *FGFR2* interacted co-essentially with *FAT1*. Indeed, *FAT1* is a biomarker of immune checkpoint blockade in lung cancer [28], and inhibition of *FGFR2* is also effective for the treatment of lung cancer [29]. The 17 cancer types with the highest modularity in the co-essentiality network were selected for further analysis in precision oncology tasks.

To investigate the robustness of the results, we leveraged a different driver gene resource, Cancer Gene Census (CGC) [30]. The modularity (*m*) of driver genes was the highest in the co-essentiality network in 12 out of the 19 cancer types (Figure S4; see Materials and methods). Additionally, we employed a distinct modularity metric, the clustering coefficient, which focuses more on link connectivity. For 16 out of the 19 cancer types, the clustering coefficient of driver genes in the co-essentiality network was higher than that in other molecular networks (Figure S5). We also explored how the modularity of driver genes is influenced by the total number of cell lines used to construct the co-essentiality network. With the inclusion of more cancer cell lines, the co-essentiality networks became more refined, revealing additional disease modules that could serve as potential therapeutic targets (Figure S6).

We further investigated whether the high modularity of co-essentiality links could be utilized to identify driver genes in each cancer type by connecting them to known driver genes based on guilt-by-association (Figure 2F). We found that co-essentiality links were more suitable than other molecular links for identifying cancer driver genes. The co-essentiality network outperformed the three other molecular networks in discovering driver genes using guilt-by-association in 15 of the 17 cancer types (Figure 2G). For example, in the case of head and neck cancer, co-essentiality links improved driver gene discovery by 17%, 16%, and 33% than other networks, respectively. Similarly, the co-essentiality network showed higher performance in 13 of the 17 cancer types compared with seven other PPI networks (Figure S7): BioPlex [31], GPSnet [7], HURI [32], InBioMap [33], iRefIndex [34], Pathway Commons [35], and STRING [36]. Furthermore, our co-essentiality network demonstrated superior performance in identifying driver genes compared to previously published networks (Wainberg_etal [11], Amici_etal [12], Gheorghe_etal_Ceres, and Gheorghe_etal_BF [37]) and a genetic interaction network based on synthetic-lethal relationships (cSLnet) [34] in 14 out of 17 cancer types (Figure S8). The co-essentiality network also surpassed a “without-network” control method that relied solely on the gene essentiality of cell lines from a specific cancer type, devoid of network support (Figure 2G, orange bars).

One might wonder if the performance of the co-essentiality network is driven by the frequency of tumor types used to construct it. We found that the ability of the co-essentiality network to identify driver genes was not significantly correlated with the number of cell lines per tumor type in the DepMap (Spearman’s correlation coefficient *rho* = 0.167, *P* = 0.522; Figure S9). Additionally, we evaluated whether the characteristics of the co-essentiality network were merely influenced by its high proportion of essential genes. Contrary to what might be expected, only 11.4% of genes in the co-essentiality network were essential, ranking it 8th among the 13 networks (Table S2).

Using two additional reported methods for driver gene identification, we found that co-essentiality links resulted in the best performance improvements and provided a robust benefit for the identification of cancer driver genes. The performance for driver gene identification was measured using network propagation methods such as Hotnet2 [38] (Figure S10A) and uKIN [39] (Figure S10B). In both methods, co-essentiality links identified driver genes better than any other molecular links. For 6 out of 11 cancer types used in the Hotnet2 study [38], the co-essentiality networks outperformed 15 other networks in identifying driver genes (Figure S10C). Compared to the co-expression network, which showed the second-highest performance, the co-essentiality network achieved significantly higher F1 scores with no significant correlation [Wilcoxon signed-rank test; *P* = 2.93 × 10^−3^; Pearson’s correlation coefficient (PCC = 0.580), *P* = 0.0613; Figure S10E and F]. Similarly, when using the uKIN algorithm, the co-essentiality network ranked among the top 3 performers in 15 of the 24 cancer types examined in the uKIN study [39] (Figure S10D). Although the Amici and colleagues’ [12] network ranked in the top 3 for 19 of the 24 cancer types, there was no significant difference between their actual areas under the receiver operating characteristic (AUROC) values (Wilcoxon signed-rank test; *P* = 0.169; Figure S10G). Furthermore, the AUROC rankings of these two networks showed an inverse correlation (PCC = −0.452, *P* = 0.0266; Figure S10H), indicating that each co-essentiality network with the uKIN algorithm exhibits distinct strengths across different cancer types. Taken together, our results suggest that the co-essentiality network captures more relationships between cancer driver genes, indicating that co-essentiality links are more relevant to cancer phenotypes than other molecular links.

### Driver modules of the co-essentiality network can differentiate patient survival

To examine the clinical relevance of the modularity of driver genes in the co-essentiality network, we evaluated whether the modules derived from these driver genes improved our understanding of cancer prognosis in terms of patient survival (Figure 2H). Among the 17 TCGA cancer types, 16 with sufficient patient survival data were tested. We found that driver modules from the co-essentiality network were more informative for stratifying patients along with overall survival than other molecular networks (Figure 2I; Figure S11). In each network, driver modules representing biological pathways in close proximity to the driver genes were tested to discern patient survival. Specifically, patients were divided into two groups based on the overall expression of the driver module, and the significance of the survival difference between the two groups was measured using the log-rank test.

The co-essentiality links provided the best driver modules stratifying patients into groups with different survival rates in 12 out of 16 cancer types. In contrast, the driver modules from other molecular networks stratified patients in 3, 11, and 5 cancer types for PPI (BioGRID), co-expression, and co-methylation networks, respectively (Figure 2I; Figure S11–14). For example, in LUSC, the pathway of signaling by non-receptor tyrosine kinases (R-HSA-9006927), which was significantly proximal to driver genes in the co-essentiality network [NES = 4.13, FDR = 9.99 × 10^−4^; Figure S16A], could stratify patients with its down-regulation, exhibiting longer overall survival than those in other groups (log-rank test, adjusted *P* = 1.75 × 10^−3^; Figure S16B and C). In contrast, co-expression and co-methylation links lined up different driver modules, Ovarian tumor domain proteases (R-HSA-5689896) and signaling pathways by FGFR2 in disease (R-HSA-5655253), respectively, and showed less significant differentiation in the overall survival of patients for co-expression (log-rank test, adjusted *P* = 0.0408; Figure S13) and non-significant difference for co-methylation (log-rank test, adjusted *P* = 0.314; Figure S14). PPIs provided no driver module because no biological pathway was significantly proximal to driver genes (Figure S12).

We also found that driver modules based on co-essentiality links were more informative for patient survival than the driver genes themselves. The control method using driver genes alone, without module identification using the co-essentiality network, was unable to differentiate survival outcomes in 15 out of 16 cancer types (bottom line of Figure 2I; Figure S15). In LUSC, for example, the patient groups stratified by expression levels of the driver module using co-essentiality links and the non-receptor tyrosine kinases pathway, showed a significant difference in the overall survival (log-rank test, adjusted *P* = 1.75 × 10^−3^; Figure S16C); in contrast, the patient groups distinguished by the expression levels of driver genes themselves showed no significant difference in overall survival (LUSC; log-rank test, adjusted *P* = 0.528; Figure S16D).

### Co-essentiality network can identify potential drug targets or biomarkers for specific cancer types

**Figure 3:**
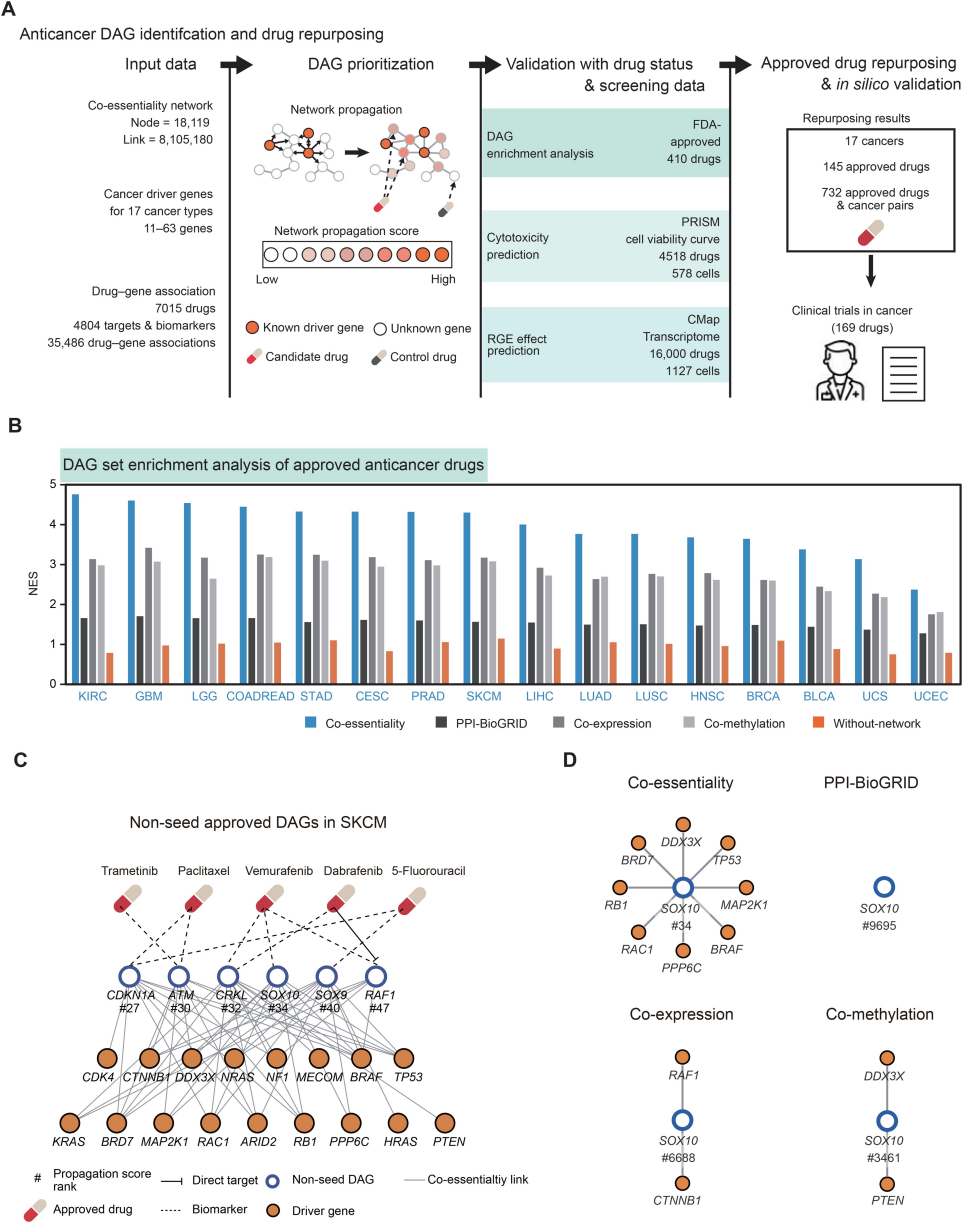
Prioritization of anticancer DAGs in 16 cancer types with available FDA-approved drug–gene associations using the co-essentiality network **A**. A schematic view of DAG prioritization using the co-essentiality network and the validation of prioritized DAGs. **B**. NES from GSEA of the four networks (co-essentiality, PPI-BioGRID, co-expression, and co-methylation), and the “without-network” control. For blue-colored cancer types, the co-essentiality network showed the highest NES among the four networks. **C**. Subnetwork of six approved DAGs (*CDKN1A*, *ATM*, *CRKL*, *SOX10*, *SOX9*, and *RAF1*) in SKCM and driver genes in their first neighbors in the co-essentiality network. Only drug–gene associations of these six genes are shown in this figure. **D**. First neighbors of *SOX10* in the four networks. NES, normalized enrichment score; GSEA, gene set enrichment analysis; DAG, drug-associated gene; RGE, reversal gene expression.

To explore the therapeutic potential of the co-essentiality network, we examined the potential of co-essentiality links for drug repurposing tasks in cancer (**Figure 3**). Using network propagation, we examined genes closely located to driver genes in the co-essentiality network. These genes may serve as targets or biomarkers for anticancer drugs [*i.e.*, drug-associated genes (DAGs)]. We utilized them for identifying new indications of approved drugs (Figure 3A). We verified the performance of the co-essentiality network in identifying therapeutic target genes by three ways: (1) prioritization of Food and Drug Administration (FDA)-approved DAGs (Table S3) [40]; (2) prediction of drug cytotoxicity on cancer cells (IC_50_); and (3) prediction of drug reversal gene expression (RGE) effects on cancer cells [41]. Finally, we validated 145 repurposing candidates predicted through co-essentiality links using cancer-related clinical trial records.

Network propagation with co-essentiality links prioritized DAGs of FDA-approved anticancer drugs better compared with 15 other networks, including other co-essentiality networks (Figure 3B, Figures S17A and  S18A). We investigated 16 out of the 17 TCGA cancer types, excluding esophageal carcinoma (ESCA), which lacks available FDA-approved DAGs. In all 16 of these cancer types, the propagation values, determined using co-essentiality links, demonstrated the highest positive enrichment for the FDA-approved DAGs. The average NES of co-essentiality links was 4.14, which was 39% higher than that of co-expression links that had the second-highest NES (Figure 3B). We also performed separate analyses for drug targets and biomarkers. For drug targets, analysis was limited to 9 out of 17 TCGA cancer types due to a lack of approved target information for the remaining types. The co-essentiality network outperformed other networks in prioritizing targets for 5 of 9 cancer types (Figures S17B, S18B, and S19A). For biomarker prioritization, the co-essentiality network demonstrated superior performance across all 15 analysable cancer types (Figures S17C, S18C, and S19B). Having greater NES to propagation values of co-essentiality links suggests that the co-essentiality network prioritizes DAGs of anticancer drugs more efficiently.

For example, in skin cutaneous melanoma (SKCM), the co-essentiality network had an NES value of 4.30, which was higher than those of PPI-BioGRID, co-expression network, and co-methylation network (1.57, 3.17, and 3.08, respectively). Among the top 50 genes with the highest propagation value from the co-essentiality network, 20 were identified as FDA-approved DAGs, which was higher than the number of PPI-BioGRID, co-expression network, and co-methylation network (18, 14, and 14 genes, respectively). Among these 20 genes, 3 (*BRAF*, *MAP2K1*, and *RAF1*) were both targets and biomarkers, and 17 (*TP53, RAC1, CTNNB1, KRAS, RB1, PTEN, NF1, NRAS, HRAS, KIT, GNA11, CDKN2A, CDKN1A, ATM, CRKL, SOX10,* and *SOX9*) were only biomarkers of approved drugs (Table S4). Similarly, in LUSC, the co-essentiality network had an NES value of 3.77, which was higher than those of PPI-BioGRID, co-expression network, and co-methylation network (1.51, 2.76, and 2.70, respectively). Among the top 50 genes with the highest propagation value from the co-essentiality network, 19 were identified as biomarkers of approved drugs (*TP53, PTEN, EP300, NF1, KDM6A, FBXW7, PIK3CA, RB1, HLA-A, FGFR2, NOTCH1, HRAS, CDKN2A, NF2, CRKL, RAC1, LATS2, TSC2,* and *TSC1*; Table S5).

Furthermore, we found that the co-essentiality network outperformed the control method that lacked network propagation (Figure 3B, orange bars). Specifically, the co-essentiality network identified six genes (*RAF1, CDKN1A, ATM, CRKL, SOX10,* and *SOX9*) due to their multiple connections with SKCM driver genes employed as initial inputs for propagation, despite not being designated as seed genes themselves (Figure 3C). RAF proto-oncogene serine/threonine-protein kinase (*RAF1*) is one of the targets of Dabrafenib, a FDA-approved *BRAF* inhibitor [42]. In the co-essentiality network, *RAF1* showed a high propagation value (1.37 × 10^−4^ and ranked 47th), because it is linked to five known SKCM driver genes: *CTNNB1, NRAS, BRAF, NF1,* and *KRAS*. Similarly, *ATM*, a biomarker of paclitaxel [43,44] and trametinib [45], is linked to eight known SKCM driver genes: *BRD7*, *MAP2K1*, *CTNNB1*, *RB1*, *ARID2*, *BRAF*, *DDX3X*, and *TP53*. Additionally, in LUSC, the co-essentiality network identified six genes (*NF2*, *RAC1*, *LATS2*, *TSC1*, *CRKL,* and *TSC2*) due to their multiple connections with LUSC driver genes (Figure S20A). The gene amplification of CRK-like proto-oncogene, adaptor protein (*CRKL*), is one of the key mechanisms of resistance to gefitinib and erlotinib [46], FDA-approved tyrosine kinase inhibitors. *CRKL* showed a high propagation value (1.97 × 10^−4^ and ranked 28th) because *CRKL* is linked to 12 known LUSC driver genes: *EP300, TP53, KEAP1, NFE2L2, RB1, PTEN, FAT1, ARHGAP35, NF1, CUL3, RASA1,* and *FBXW7*. These results represent the advantages of utilizing the co-essentiality network approach over gene-centric approaches without a network.

We observed that co-essentiality links could identify the approved DAGs that were not captured by other molecular links. For example, in SKCM, a biomarker of Vemurafenib [47], *SOX10*, ranked 34th in the co-essentiality network, but 9695th in the PPI network, 6688th in the co-expression network, and 3461st in the co-methylation network (Figure 3D). The co-essentiality network could identify more interactions between *SOX10* and known driver genes of SKCM that were not captured in other molecular links. *SOX10* had co-essentiality links with seven driver genes of SKCM: *TP53, MAP2K1, BRAF, PPP6C, RAC1, RB1*, and *BRD7*, which were not connected to other molecular networks. A biomarker of three approved drugs (everolimus, carboplatin, and erlotinib), *NF2* ranked 25th in the co-essentiality network, but only 476th in the PPI network, 8975th in the co-expression network, and 3408th in the co-methylation network (Figure S20B). The co-essentiality network identified multiple interactions between *NF2* and known driver genes of LUSC that were not captured in other molecular networks. Specifically, *NF2* demonstrated co-essentiality links with five driver genes of LUSC: *RB1*, *CUL3*, *NF1*, *PTEN*, and *KEAP1*, connections that were absent in the other molecular networks examined. This suggests that co-essentiality links can facilitate the identification of new DAGs that are not covered by other molecular links.

### Co-essentiality network finds candidates for drug repurposing

**Figure 4:**
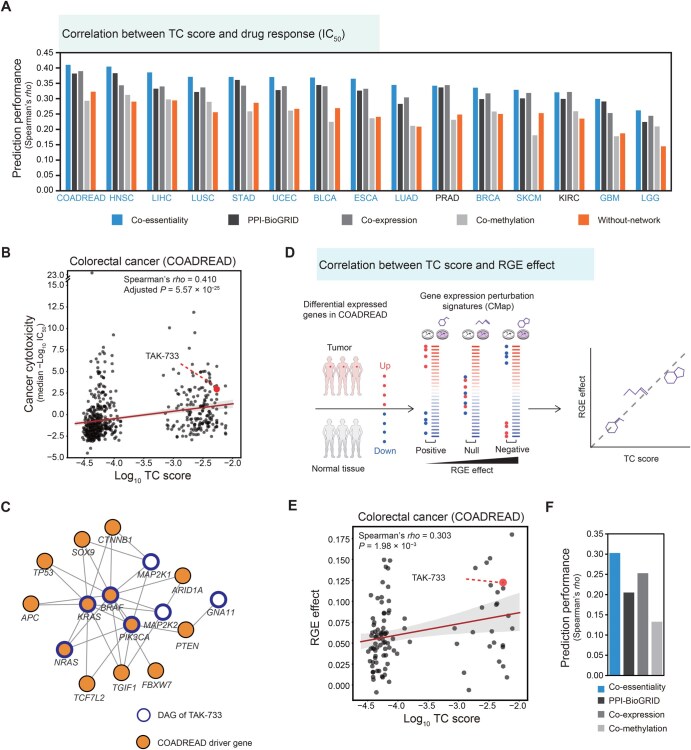
Predicting drug response in cancer cells of 15 cancer types using therapeutic candidate score of the co-essentiality network **A**. Prediction performance of drug response measured using the Spearman’s correlation coefficient between TC score and median −log_10_ IC_50_ for the four networks (co-essentiality, PPI-BioGRID, co-expression, and co-methylation), and the “without network” control. For blue-colored cancer types, the co-essentiality network showed the best performance among the four networks. **B**. Scatter plot of drug response and TC score of COADREAD in the co-essentiality network and data point of candidate drug: TAK-733 (red dot). **C**. A subnetwork of seven DAGs of TAK-733 and COADREAD driver genes connected in the co-essentiality network. **D**. Schematic diagram of drug RGE effect on COADREAD. **E**. Scatter plot of drug’s RGE effect and TC score of COADREAD in the co-essentiality network. The red data point indicates a candidate drug, TAK-733. **F**. Performance of the four networks for predicting drug RGE effect in COADREAD. TC, therapeutic candidate.

Having confirmed the capability of co-essentiality links to find DAGs of approved drugs, we further investigated whether they could facilitate network-based prediction of drug responses assessed in large-scale pharmacogenomic screenings (**Figure 4**). For each drug, we assigned a therapeutic candidate (TC) score aggregated from the propagation value of its DAGs by considering their root mean square (RMS). Therefore, we considered the average effect of DAGs on specific cancer types to eliminate the bias.

We found that the TC score calculated from co-essentiality links predicted the cytotoxicity of cancer drugs better than scores derived from other molecular links. For each cancer type, drugs with high TC scores from the co-essentiality links were highly cytotoxic to cancer cell lines of that cancer type. Co-essentiality links showed the highest correlation between TC scores and the IC_50_ values in 13 out of 15 cancer types with drug screening data (Figure 4A), and the average increase in the Spearman’s correlation coefficient (Spearman’s *rho*) was 9.7%, 8.4%, and 43% for the PPI, co-expression, and co-methylation links, respectively. In colorectal cancer (COADREAD; Spearman’s *rho* = 0.410, adjusted *P* = 5.54 × 10^−25^), for example, TAK-733, an MEK1/2 inhibitor under clinical trials for advanced non-hematologic malignancies and advanced metastatic melanoma [48,49], was predicted to have an antitumor effect (Figure 4B; TC score = 5.39 × 10^−3^, rank percentile = 98.57%), and indeed showed high cytotoxicity in the cancer cell lines of COADREAD (median IC_50_ = 1.03 × 10^−3^ µM). In the co-essentiality network, TAK-733 had seven DAGs: *MAP2K1, MAP2K2*, *BRAF, KRAS, NRAS, PIK3CA,* and *GNA11* (Figure 4C, blue circles), which were interconnected by nine driver genes of colorectal cancer, namely *CTNNB1*,* SOX9*,* TP53*,* APC*,* TCF7L2*,* TGF1*,* FBXW7*,* PTEN*, and *ARID1A* (Figure 4C, orange filled cycles). For LUSC, Everolimus, an mTOR kinase inhibitor approved for various malignancies, was predicted to have an antitumor effect (Spearman’s *rho* = 0.371, adjusted *P* = 8.27 × 10^−21^). This prediction (Figure S21A; TC score = 2.94 × 10^−3^, rank percentile = 93.74%) was validated by the high cytotoxicity of Everolimus demonstrated in LUSC cancer cell lines (median IC_50_ = 4.47 × 10^−4^ µM). In the co-essentiality network, Everolimus had 36 DAGs (Figure S21B, blue circles), which were interconnected by 21 driver genes of LUSC (Figure S21B, orange filled cycles).

Similarly, the co-essentiality network showed higher correlations between the TC scores and IC_50_ values in 12 out of 15 cancer types than the other 7 PPI networks (Figure S22). Similarly, the co-essentiality network showed higher correlations than other co-essentiality networks and a genetic interaction network in 8 out of 15 cancer types (Figure S23).

One might question whether driver genes alone, without the assistance of network propagation, can predict drug responses. We found that the control method using driver genes alone was not as predictive as the TC score of the co-essentiality network (Figure 4A, orange bars). The TC scores showed greater correlations with IC_50_ values for all 15 cancer types than driver genes, and the average increase in Spearman’s *rho* was 42%. This is because driver genes cover only 4.4% of drug targets, whereas using network propagation, all the genes in networks were assigned propagation scores.

Additionally, we assessed whether incorporating biomarker information enhances the accuracy of drug response predictions. The TC score using DAGs, including both target and biomarker information, showed a greater correlation with IC_50_ values across 15 cancer types than using only target information, and the average increase in Spearman’s *rho* was 32.1% (Figure S24). This result indicates that leveraging both drug targets and biomarkers improves the prediction of the anticancer effects of drugs, compared to relying solely on direct target data.

Focusing on COADREAD, which showed the greatest correlation between TC score and IC_50_ value, we further discovered that drugs with high TC scores could alter gene expression, reverting it to levels observed in cancer conditions (Figure 4D). We used the degree of reversal effect of the drugs on cancer-associated gene expression to confirm the validity of the TC score, since a drug capable of ameliorating the perturbed molecular state induced by a disease exhibits potential for therapeutic efficacy. We measured the RGE effect [50], which is the dot product of ranked differential expression between cancer [51] and drug-treatment conditions [52] (Figure 4D), and investigated its association with the TC score. We observed that the TC score of the co-essentiality network was positively correlated with the RGE effect (Figure 4E; Spearman’s *rho* = 0.303, *P* = 1.98 × 10^−3^). For example, TAK-733, which exhibited a high TC score and −log_10_ IC_50_ in Figure 4B, showed a strong reversal effect on the expression pattern (RGE effect = 0.123, rank percentile = 91.18%). Additionally, the TC score from the co-essentiality network exhibited a stronger correlation with the RGE effect than other molecular networks (Figure 4F). Overall, these results indicate that the co-essentiality network is more effective than the molecular networks based on PPI, co-expression, and co-methylation for identifying driver-associated therapeutic targets, which holds potential for drug repurposing.

### *In silico* drug repurposing of approved drugs using the co-essentiality network

**Figure 5:**
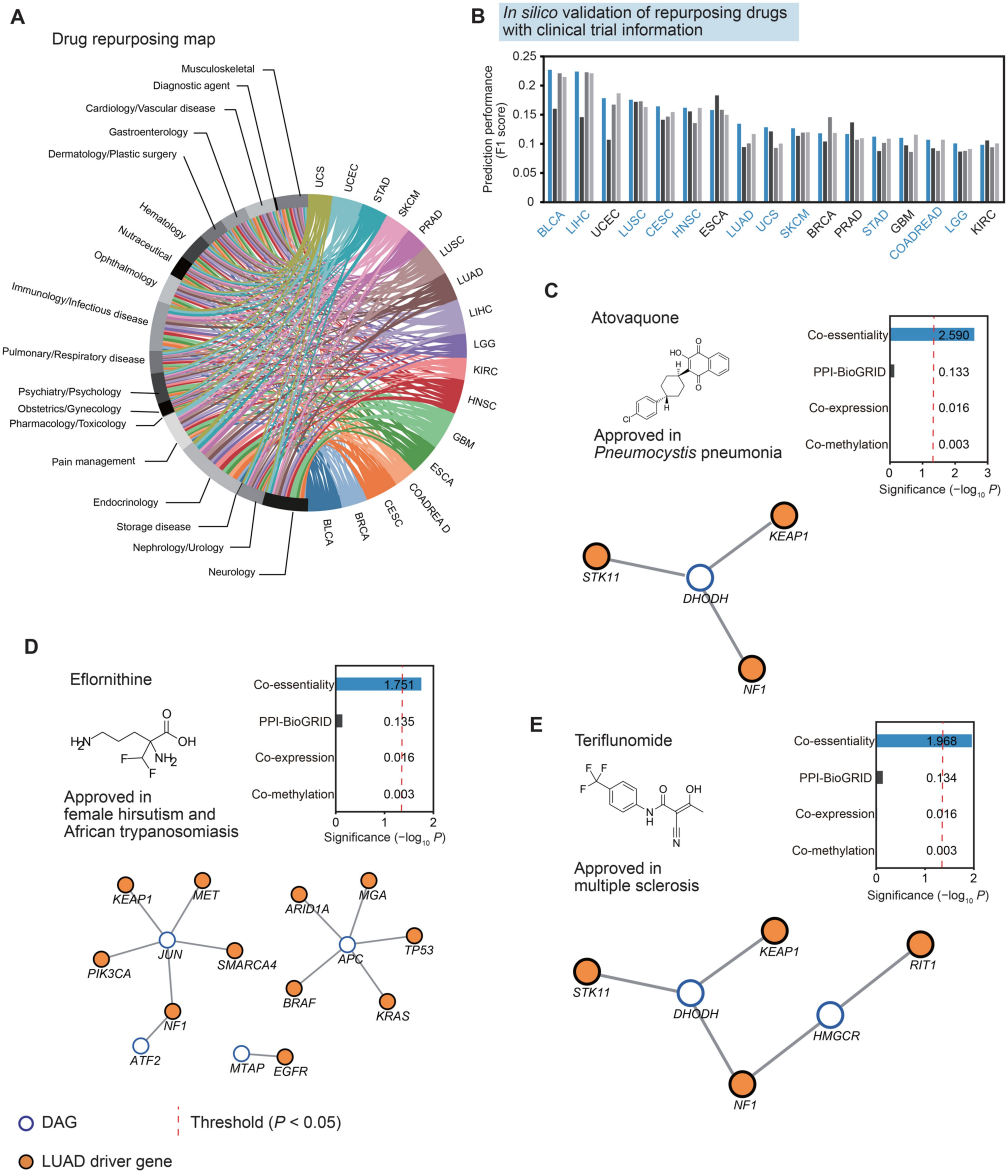
*In silico* drug repurposing using co-essentiality network and case studies in lung adenocarcinoma **A**. A chord plot illustrating a global view of potential anticancer indications for 145 approved drugs across 17 cancer types. **B**. Validation of the drug’s new indication using clinical trial records. The prediction performance of drug indication is measured with the F1 score. For blue-colored cancer types, the co-essentiality network showed the best performance among four networks: co-essentiality, PPI-BioGRID, co-expression, and co-methylation. **C**.–**E**. Drug repurposing candidates in LUAD predicted by the co-essentiality network but not by other networks. The bar graphs show the significance of the TC score, and the red dashed lines show the threshold of drug repurposing (adjusted *P* = 0.05). Below, a subnetwork of DAGs and LUAD driver genes is connected to them in the co-essentiality network.

To identify the novel therapeutic potential of the approved drugs for other purposes, we conducted *in silico* drug repurposing using the TC score from the co-essentiality network (**Figure 5**). Among a total of 1213 approved non-cancer drugs having drug–gene associations from the PanDrugs database [53], 145 were predicted to have significant anticancer activities in at least one of the 17 cancer types by the co-essentiality network (adjusted *P* < 0.05; Figure 5A; Table S6). To identify repurposing candidates, we measured the significance of the TC score of drugs from a permutation test in each cancer type (See Materials and methods). For example, pioglitazone hydrochloride had a TC score of 1.39 × 10^−3^ in lung adenocarcinoma (LUAD), and the significance of this score, calculated from the permutation test of random DAGs, is adjusted *P* of 3.79 × 10^−3^ (Figure S25).

To further assess the effectiveness of *in silico* drug repurposing utilizing the co-essentiality network, we explored whether the repurposing candidates had clinical trial records related to cancer. For 11 out of the 17 cancer types analyzed, the repurposing results from the co-essentiality network demonstrated enhanced performance in predicting drugs with clinical trials for cancer, compared to those from PPI-BioGRID, co-expression, and co-methylation networks (Figure 5B). Similarly, the co-essentiality network showed higher performance in 6 cancer types than the 7 other PPI networks (Figure S26). In addition, the co-essentiality network performed better than four other co-essentiality networks and a genetic interaction network across 7 cancer types (Figure S27).

Furthermore, we demonstrated that our co-essentiality network-based repurposing method outperformed an existing network-based approach presented by Cheng and colleagues [16], which has been widely applied across multiple diseases [54,55]. The method relies on network proximity between disease genes and drug targets to generate drug repurposing prediction scores. Across all 17 cancer types analyzed, our co-essentiality network showed superior performance in predicting drugs with cancer-related clinical trials compared to the method reported by Cheng and colleagues, regardless of which proximity threshold (*z*) was applied (Figure S28).

Specifically, we identified 30 non-cancer drugs with new indications not addressed by other networks. In the case of LUAD, we identified three repurposing candidates (atovaquone, eflornithine, and teriflunomide) that were solely predicted by the co-essentiality network, distinct from other networks, and interestingly, two of these drugs were associated with documented clinical trials in cancer (Table S7). For example, atovaquone, a hydroxynaphthoquinone, is approved for Pneumocystis pneumonia and was predicted to have a significant antitumor effect on LUAD (*P* = 2.57 × 10^−3^; Figure 5C). Atovaquone inhibits a mitochondrial protein, dihydroorotate dehydrogenase (*DHODH*), which has co-essentiality links with three LUAD driver genes (*STK11*, *KEAP1*, and *NF1*), resulting in inhibition of the electron transport chain and a loss of mitochondrial function. Several studies have described the anticancer effect of atovaquone [56,57], and there are two clinical trials of atovaquone in non-small-cell lung cancer (NCT04648033 and NCT02628080).

Another repurposing candidate, eflornithine, an ornithine decarboxylase (*ODC1*) inhibitor indicated to treat female hirsutism and African trypanosomiasis, was exclusively identified by the co-essentiality network (*P* = 0.0177; Figure 5D). Although in the co-essentiality network, *ODC1* had no connection with driver genes of LUAD, four biomarkers (*JUN*, *APC*, *ATF2*, and *MTAP*) showed links with 11 LUAD driver genes. This is also supported by previous literature [58,59] and clinical trials (NCT05500508).

Finally, the co-essentiality network identified teriflunomide, a pyrimidine synthesis inhibitor used to treat relapsing multiple sclerosis, as potentially effective for LUAD (adjusted *P* = 0.0109; Figure 5E). Two targets of the drug, *DHODH* and 3-hydroxy-3-methylglutaryl-CoA reductase (HMGCR), have co-essentiality links with four LUAD driver genes (*STK11*, *KEAP1*, *RIT1*, and *NF1*). Furthermore, the antitumor effect of HMGCR inhibition has been demonstrated in preclinical studies using either fluvastatin [60] or CRISPR knockout [61]. Although teriflunomide lacks direct clinical trial evidence for LUAD, this prediction is supported by preclinical studies examining its effects in non-small cell lung cancer (NSCLC) cell lines and mouse xenograft models [62]. These findings suggest that network propagation with co-essentiality links can identify promising candidates for drug repurposing.

## Discussion

In this study, we showed that therapeutic targets of cancer could be identified using co-essentiality links (Figure 3). We applied a co-essentiality network to systematically prioritize anticancer targets and biomarkers in various cancer types and demonstrated that it performed better than other molecular networks, including PPI, co-expression, and co-methylation networks (Figure 3A). We constructed a co-essentiality network using phenotype information, where links were inferred from correlations of gene essentiality profiles of cancer cells (Figure 1). Since gene essentiality is measured by the proliferation rates of cancer cells, a crucial indicator of anticancer therapy, gene essentiality profiles have been applied to identify promising targets of anticancer therapies [19]. Gene essentiality was also utilized to form a network. For example, co-essentiality networks have been built to identify a group of genes with the same functional complexes [14] or metabolic pathways [15] and new functions of genes [10–13,63]. However, for therapeutic purposes, co-essentiality networks have not been applied to identify the target genes of anticancer therapies. Here, we demonstrated that links in the co-essentiality network could capture functional pathways related to the proliferation of cancer cells (Figure 1C and D). Our results suggested that co-essentiality links can prioritize DAGs for cancer treatment.

Since the network approaches are dependent on the topology of the networks, finding a suitable network is crucial for developing network medicine [8,9]. Here, we observed that the co-essentiality network showed an improvement in driver gene identification when compared with diverse molecular networks (Figure 2G). Using well-known network propagation approaches, such as Hotnet2 [38] and uKIN [39], our co-essentiality network showed improved prediction performance in identifying driver genes compared with other molecular layered networks (Figure S10). Specifically, the co-essentiality network showed higher modularity between driver genes than any other molecular network (Figure 2A, Figures S3 and S4). This topological characteristic of the co-essentiality network contributes to the high performance of the driver gene identification. In fact, the modularity of disease genes in specific networks may be the key to identifying disease genes in various diseases [9]. Thus, for precision oncology, the choice of a network with highly clustered cancer driver genes can improve task performance.

It is essential to note that for each purpose, one may need to employ a customized approach to obtain an optimal network. For drug repurposing, we demonstrated that the co-essentiality network developed here performed better than other networks, including other co-essentiality networks (Figures S18, S23, and S27). Since the repurposing process relies on network propagation, our co-essentiality network would take advantage of a greater number of direct links than other networks, with much less loss of information during the propagation process. This observation aligns with earlier findings that the performance of disease gene identification is positively correlated with the size of a network [8]. Specifically, these direct links likely embody the phenotypic associations recorded from CRISPR-Cas9 screening into topological paths between the target and seed nodes [64]. In contrast, when aiming to discover novel biochemical interactions, such as metabolic pathways [15] or protein complexes [14], it would be more relevant to obtain smaller co-essentiality networks in which links have a greater chance to represent such molecular interactions with fewer false positives. Given a specific purpose, it remains a crucial scientific challenge to identify an appropriate approach to building biological networks with varying link properties and topological features [9,61].

For drug repurposing tasks, we found that drug–gene associations that include both targets and biomarkers provided more benefit for predicting the drug’s therapeutic potential. A “biomarker” is a gene whose genetic status is linked to treatment response (based on pre-clinical or clinical evidence), but the protein product itself is not the pharmacological target. However, numerous studies have highlighted the potential for biomarkers to become druggable and the ability to guide the appropriate drug–disease associations [53,65]. We discovered that utilizing drug–gene associations, which include both targets and biomarkers, improves the precision in predicting the anticancer effects of drugs compared to using only direct target data alone (Figure S24). Furthermore, our study identified eflornithine as a potential candidate for repurposing, due to its four biomarkers (*JUN*,* APC*,* ATF2*, and *MTAP*; Figure 5) being connected to driver genes of LUAD. These cases suggest that including biomarker information could enhance the effectiveness of network medicine.

We found that the quality of the co-essentiality network depended on the number of cell lines whose essentiality profiles were utilized to build co-essentiality links. As more cancer cell lines were compiled, co-essentiality networks improved and identified additional disease modules that serve as potential therapeutic targets (Figure S6). Although gene essentiality data of cancer cells have been continuously accumulated in 769 cancer cell lines through various studies [66,67], the number of currently available cancer cell lines with measured gene essentiality profiles does not appear to be sufficiently saturated to establish complete co-essentiality links for precision oncology. Thus, the co-essentiality network has room for improvement with an increase in the number of cell lines in which essentiality profiles are currently being measured. Therefore, gene essentiality profiling of a more diverse set of cancer cells is essential to improve the capacity of resources to define a more suitable network for precision oncology.

Like any other network-based *in silico* drug repurposing method, our co-essentiality network-based method has several limitations that should be acknowledged: (1) While our co-essentiality network effectively prioritized anticancer targets and drug repurposing candidates, the network is based on data from cancer cell lines, which may not fully recapitulate the complexity of tumor biology *in vivo*. Future work could incorporate additional data from patient-derived models or *in vivo* studies to refine the network. (2) Our drug repurposing predictions were validated using clinical trial data, but further experimental validation in preclinical models would strengthen the evidence for these candidates. (3) Our network focuses on protein-coding genes, but non-coding RNAs and epigenetic factors also play important roles in cancer biology. Integrating these additional layers of biological complexity could further enhance the predictive power of the network. Despite these limitations, our study provides a valuable resource and framework for leveraging co-essentiality networks in precision oncology, and we anticipate that future work will build upon and extend our findings.

## Materials and methods

### Resources and network information

For all 16 networks used in this study, nodes were converted to HUGO symbols, while only the nodes and links included in the largest connected component were selected. The number of nodes and links of the final networks used in this study is reported in Table S2.

### Genome-wide data for network construction

#### Gene essentiality data

To build a co-essentiality network, we used a dataset consisting of the genome-wide CRISPR screening data from the Achilles project 20q2 in the dependency map (DepMap) project [19]. Specifically, the dataset was an 18,119 × 769 matrix of gene essentiality, including 18,119 genes, which were screened in 769 cell lines from 26 distinct lineages using the Avana CRISPR library [68]. The dataset can be downloaded from https://depmap.org/portal/download/all/ by selecting the “DepMap Public 20q2” release and the “Achilles_gene_effect.csv” file.

#### Gene expression data

To construct a co-expression network, we used CCLE expression data quantified from RNA-seq files using GTEx pipelines [69]. The dataset contains the gene expression data of 19,144 genes in 1305 cell lines from 34 distinct lineages. Among the 19,144 genes, 23 with zero expression values across the cell lines were removed. The dataset can be downloaded from https://depmap.org/portal/download/all/ by selecting the “DepMap Public 20q2” and the “CCLE_expression_v2.csv” file.

#### Gene methylation data

To construct a co-methylation network, we used CCLE DNA methylation reduced representation bisulfite sequencing data (promoter 1 kb upstream of transcription start site) [69]. The dataset consists of the methylation data of 21,337 loci covering 17,182 gene promoter regions in 843 cell lines. Due to the many missing values in the data matrix, we retained only cell lines with methylation data of at least 17,000 loci and loci with methylation data in at least 644 cell lines. Finally, we incorporated 20,198 methylation loci from 805 cell lines into the network construction steps. The dataset can be downloaded from https://depmap.org/portal/download/all/ by selecting the “CCLE 2019” and the “CCLE_RRBS_TSS1kb_20181022. txt” file.

### Constructing the gene correlation-based network

To construct three networks (a co-essentiality network, a co-expression network, and a co-methylation network) from the corresponding dataset, we measured the similarity in essentiality, expression, and methylation between two genes using corrected correlation and used this value as the link weight in the network. After data correction, we employed link filtering steps using PCC and CLR algorithm [20], as utilized by do Valle and colleagues [70], and filtered out links that exhibited a link weight of 0. This procedure consists of three steps: (1) For all missing values, we conducted a *k*-nearest neighbor (KNN) imputation with *k* = 10 using the ‘impyute’ Python module. (2) To measure the correlation between genes, we calculated the PCCs for all pairs of genes in the datasets and used the absolute values of PCCs to capture both directions of correlations between genes. (3) For the absolute value of PCCs, we applied the CLR algorithm, which conducts adaptive background correction to eliminate false correlations and indirect influences. In particular, the PCC value between gene *i* and *j*, *r_ij_* was adjusted with the score ${\mathrm{CLR}}_{ij}=\sqrt{z_{ij}^{2}+z_{ji}^{2}}$ where zij={rij-μiσi, rij-μiσi≥t0, rij-μiσi<t, t is the threshold value for the $z_{ij}$ and $z_{ji}$. The $\mu_{i}$ and $\sigma_{i}$ are, respectively, the sample mean and standard deviation of the empirical distribution of $r_{ik}$, *k* = 1, …, *n* (*n* is the number of genes). To determine the optimal threshold *t* for filtering links in the three correlation-based networks (co-essentiality, co-expression, and co-methylation), we evaluated driver gene identification performance across networks constructed using six threshold values ranging from 0.0 to 5.0 (Figure S29; Table S8). We selected *t* = 2.0 as this threshold because it yielded optimal performance for co-expression and co-methylation networks in identifying cancer driver genes, while maintaining robust performance for the co-essentiality network.

One could argue that for the co-expression network, employing a non-parametric method like the maximal information coefficient (MIC) is more suitable than a parametric method such as the PCC, given that gene expression often deviates from a normal distribution. To address this, we compared the efficacy of PCC-based and MIC-based co-expression networks in identifying cancer driver genes. At a threshold of *t* = 2.0, where both networks demonstrated their peak performance, the PCC-based network surpassed the MIC-based network in terms of effectiveness (Table S9). Consequently, we selected for the PCC-based co-expression network.

### Preparation of PPI network

Eight human PPI networks were used: BioGRID [25], BioPlex [31], GPSnet [7], HURI [32], Inbiomap [33], iRefIndex [34], Pathway Commons [35], and STRING [36]. For all PPI networks, the link weight values were set to 1.0.

#### BioGRID

We downloaded the BioGRID interactome from https://thebiogrid.org/ under BIOGRID-4.1.190. We used the interactions between both proteins from *Homo sapiens*.

#### BioPlex

We downloaded the following BioPlex interactomes from https://bioplex.hms.harvard.edu/interactions.php: BioPlex 3.0 Interactions (293 T cells) and BioPlex HCT116 (v1.0) (HCT116 cells). We constructed a single network from the union of both interactomes.

#### GPSnet

We used the GPSnet interactome previously constructed by Cheng and colleagues [7], which assembled 15 commonly used databases with multiple experimental sources of evidence and an in-house systematic human PPI. The interactome is publicly available at https://github.com/ChengF-Lab/GPSnet/tree/master/Data_mat and file “Net_PPI.mat”. The GPSnet was originally an *in silico* framework for drug repurposing, and in this study, we called the PPI network in this framework.

#### HURI

We downloaded the HURI interactome from http://www.interactome-atlas.org/ and the file “HuRI.tsv”.

#### InBioMap

We downloaded the InBioMap interactome from https://zs-revelen.com/download and the file “InBio_Map_core_2016_09_12”.

#### iRefIndex

We downloaded the iRefIndex interactome from the web interface to the Interaction Reference Index repository (iRefWeb, https://wodaklab.org/iRefWeb/) under the release iRefIndex (v13.0). We used four searching options: “single organism interaction”, “*Homo Sapiens*”, “experimental”, and “physical”.

#### PathwayCommons

We downloaded the PathwayCommons interactome from http://www.pathwaycommons.org/archives/PC2/v12/ and the file “PathwayCommons12.All.hgnc.txt.gz”.

#### STRING

We downloaded the STRING interactome from https://string-db.org/ under the release v11.0. To avoid co-citation information in STRING, we removed the text-mining scores for all links and recalculated the confidence score of STRING. Next, links with confidence scores > 700 were considered to leverage the high-confidence PPIs.

### Benchmark of co-essentiality networks

Four co-essentiality networks from three published studies were used as benchmarks for our approaches. We reconstructed each network using the same gene essentiality dataset employed in our co-essentiality network construction (18,119 genes × 769 cell lines) to eliminate performance bias from different cell line compositions. The first benchmark network, constructed by Wainberg and colleagues [11], was built using generalized least squares correlation. The second network, constructed by Amici and colleagues [12], was constructed using a rank-based bottom-up approach with a rank threshold of 30, as selected in the original publication. The remaining two networks from Gheorghe et al. [37], hereafter referred to as Gheorghe et al.–Ceres and Gheorghe et al.–BF, were generated using the principal component analysis (PCA) whitening method, but differed in their essentiality measurements used: the former utilized pre-calculated Ceres scores from DepMap, whereas the latter used Bayes Factors calculated using BAGEL2. The networks were constructed following each study’s methods and source code.

### Genetic interaction network

To compare the co-essentiality network with the genetic interaction network based on a synthetic lethal relationship, we used a clinically relevant synthetic lethality network built by the “identification of clinically relevant synthetic lethality (ISLE)” approach [71]. We downloaded the “clinically relevant synthetic lethality network (cSLnet)” interactome from https://github.com/jooslee/ISLE/tree/main/networks and the file “ISLE_clinical_SL_network_FDR_0.2.cys”.

### Essential gene fraction in the networks

To measure the fraction of essential genes in the network, we used the 2123 common essential genes in the DepMap. The dataset is publicly available at https://depmap.org/portal/download/all/ under the release “DepMap Public 20q2” and file “Achilles_common_essentials.csv”.

### GSEA of co-essentiality network

We calculated the enrichment of co-essentiality links in six curated gene sets. We downloaded six curated gene sets: molecular pathways (KEGG [21], Reactome [23]) and GO annotations (BP, MF, and CC) [24] from Molecular Signatures Database (MSigDB) [72], and human core protein complexes from CORUM [22].

In the co-essentiality network, gene pairs were ranked by the link weights and grouped into cumulative bins of 10,000 pairs, and the enrichment was calculated using the ratio of pairs annotated with the same biological modules. We also measured enrichment expected by chance as the probability of finding the gene pairs within the same biological modules without being informed by co-essentiality links. Similar to the approach of Lee and colleagues [73], for the expected ratio, we changed the denominator from the number of co-essentiality links to all possible pairs between the genes given a bin.

### Modularity calculation

We used two types of modularity measures as shown in the following function: $\mathrm{cohesiveness}(G)=\frac{W_{in}}{W_{in}+W_{\mathrm{out}}},\;$where $W_{in}$ is the total weight of links exclusively present within group of genes *G*, $W_{\mathrm{out}}$ is the total weight of links that connect the gene group with the rest of the network [74] and $\mathrm{clustering}\;\mathrm{coefficient}(G)=\frac{1}{n}\sum_{v\in G}\frac{2T_{v}}{k_{v}(k_{v}-1)}$, where n is the number of the gene group *G*, $T_{v}$ is the number of triangles through node, and $k_{v}$ is the degree of node *v*. Since both modularity measures can be affected by the degree centrality of genes in a network, we applied normalization to the modularity measures to remove the degree bias. Similar to the approach of Guney and colleagues [75], we created a reference modularity distribution corresponding to the expected modularity of 100 randomly selected groups of genes matching the size and degree distributions of query genes in the network. Next, modularity was normalized as the Z-scores of the observed modularity of genes computed from the reference distribution of modularity from random groups.

### Network enrichment to cancer-related pathways

From the complete set of 186 KEGG pathways available in MSigDB, we defined 31 pathways as CRPs. These CRPs include pathways categorized under “Pathways in cancer” (KEGG Pathway ID: hsa05200; https://www.genome.jp/entry/hsa05200; “Related pathway sections”) and all pathways classified under the KEGG subcategory “6.2 Cancer: specific types” (https://www.genome.jp/kegg/pathway.html) in the KEGG pathway database hierarchy. A comprehensive list of these 31 CRPs, along with their attributes, is provided in Table S1.

For 186 KEGG pathways, we calculated relative modularity by converting scaled modularity using cohesiveness to rank percentile scores in four networks: the co-essentiality network, PPI network (BioGRID), co-expression network, and co-methylation network. To evaluate the enrichment of CRPs in each network in an unbiased manner, we used the median relative modularity value as a threshold to classify pathways as network-specific (high modularity) or non-network-specific (low modularity). This approach ensures a balanced division of pathways while accounting for network-specific modularity distributions. The relative modularity values are presented in Table S1.

Finally, for each network, we constructed a 2 × 2 contingency table with four types of pathways, namely network-specific CRPs, non-network-specific CRPs, network-specific non-CRPs, and non-network-specific non-CRPs. From the contingency table, we calculated the odds ratio of network enrichment to CRPs and determined the statistical significance of enrichment by calculating the *P* value from Fisher’s exact test. The contingency tables of the four networks are shown in Table S10.

To further validate our findings using a threshold-independent approach, we performed GSEA with the GSEApy Python module. For each network, we ranked all 186 pathways based on their relative modularity values in descending order, creating a pre-ranked list. We then assessed whether CRPs were significantly enriched toward the top of these ranked lists. This analysis evaluated the distribution of CRPs across the entire spectrum of modularity values without imposing arbitrary thresholds. The enrichment score (ES) reflected the degree to which CRPs were overrepresented at the top of the ranked list, with the NES accounting for differences in gene set size and correlation between pathways. Statistical significance was determined through 1000 permutations. Additionally, we performed pairwise statistical comparisons of the relative modularity distributions between networks using the Wilcoxon signed-rank test. Both analyses are presented in Figure S2.

### Driver genes of each cancer type

#### Driver gene retrieval

We retrieved driver genes of each cancer type from the study by Bailey and colleagues [76], who reported 299 driver genes across 33 cancer types. For modularity analysis, we selected 19 cancer types with more than 10 reported driver genes included in the co-essentiality network: bladder urothelial carcinoma (BLCA), breast invasive carcinoma (BRCA), cervical squamous cell carcinoma and endocervical adenocarcinoma (CESC), COADREAD, lymphoid neoplasm diffuse large B-cell lymphoma (DLBC), ESCA, glioblastoma multiforme (GBM), head and neck squamous cell carcinoma (HNSC), kidney renal clear cell carcinoma (KIRC), acute myeloid leukemia (LAML), brain lower grade glioma (LGG), liver hepatocellular carcinoma(LIHC), LUAD, LUSC, prostate adenocarcinoma (PRAD), SKCM, stomach adenocarcinoma (STAD), uterine corpus endometrial carcinoma (UCEC), and uterine carcinosarcoma (UCS).

#### CGC

For further validation of the high modularity of driver genes in the co-essentiality network, we used the experimentally validated cancer driver gene set from CGC database [30]. For cancer type-specific analysis, we manually mapped Tier 1 driver genes in CGC to TCGA cancer types according to their tumor type information. A total of 470 driver genes were mapped to 22 TCGA cancer types. Similarly, for modularity analysis, we selected 19 cancer types with more than 10 reported driver genes included in the co-essentiality network: BLCA, BRCA, colon adenocarcinoma (COAD), DLBC, HNSC, kidney chromophobe (KICH), KIRC, LAML, LGG, LIHC, LUAD, LUSC, ovarian serous cystadenocarcinoma (OV), pancreatic adenocarcinoma (PAAD), PRAD, SKCM, STAD, thyroid carcinoma (THCA), and UCEC. The driver genes mapped to cancer types are listed in Table S11.

### Driver gene prediction with guilt-by-association

To evaluate the performance of each network for driver gene identification, we conducted leave-one-out cross-validation with driver genes from Bailey and colleagues [76]. For each network, we measured the prediction score of each gene via the ratio of the driver gene number in the first neighbors of the gene as follows: $S_{g}=\frac{\sum W_{\mathrm{driver}}}{\sum W_{\mathrm{all}}}$, where $S_{g}$ is the prediction score of gene *g*, $W_{\mathrm{driver}}$ is the weight of links from gene *g* connected to driver genes, and $W_{\mathrm{all}}$ is the weight of all links from gene *g*. The performance of the network was measured using the AUROC area under the curve on the prediction score, which was designed to obtain a high value when driver genes were assigned a high prediction score.

For the “without-network” control method, we calculated the prediction score using the average gene essentiality value of cell lines within the same cancer type. Using this prediction score, we measured the performance of driver gene identification using the same AUROC metric as applied in network-based approaches.

### Driver gene prediction with Hotnet2

To evaluate the performance of driver gene identification for each network, we also used a network propagation method, Hotnet2 [38], which is an algorithm for identifying significantly mutated subnetworks [38]. As inputs for HotNet2, we employed (1) the mutation data of 11 cancer types used in the original paper of HotNet2 and (2) the networks used in this study, including the co-essentiality network. The mutation data were pre-processed using the same process described in the original paper. The parameters for the permutation numbers were set to 100 for the networks and 1000 for the heat. The insulating parameter β was set to 0.04. The default settings for other parameters were used. The source code of the Hotnet2 algorithm of the original authors [38] at a GitHub repository (https://github.com/raphael-group/hotnet2) was used.

The performance of Hotnet2 was measured with the number of driver genes included in the modules resulting from Hotnet2 using the F1 score. For each cancer type, the driver genes from Bailey and colleagues [76] were used as a positive set of predictions. The F1 score was calculated as follows:


$$F1\;\mathrm{score}=2\;\times \;\frac{\mathrm{Precision}\times \mathrm{Sensitivity}}{\mathrm{Precision}+\mathrm{Sensitivity}\;}$$

### Driver gene prediction with uKIN

To test the performance of driver gene identification for each network, we used a guided network propagation method, uKIN [39], which uses prior knowledge of cancer genes to guide, within networks, the network propagation that starts from newly identified candidate genes. We built an in-house script for the uKIN algorithm, which is available at https://github.com/SBIlab/CESnet-repurposing.

Adapting the guided propagation concepts from uKIN, we conducted two stages of network propagation using the page-rank algorithm from the NetworkX Python module [77]. In the first stage, we used 1 for genes from prior knowledge in the network and 0 for all other genes in the network as an input for the personalization parameter in the page-rank algorithm. From the results of the network propagation, we redevised the network as a directed graph and re-assigned link weights as follows: $w_{ij}=\frac{p_{j}}{\sum_{k\in N(i)}p_{k}}$ where $w_{ij}$ is the weight of the link from node *i* to node *j*, $p_{k}$ is the propagation score of node *k*, and N(i) are the neighbors of node *i*. In the second stage, we conducted network propagation with a re-devised network, using new information as seeds. All other page-rank algorithm parameters were adjusted to their default values (damping factor = 0.85).

The performance of the guided network propagation was evaluated using the same method as in the uKIN study. We used 723 CGC genes as the prior and positive sets, respectively [30]. First, we separated the CGC genes that occurred in our network into two categories, namely prior knowledge set and hidden set, which indicated the seed genes for the first propagation and test set for the uKIN outcome, respectively. To create the group of positives that we hoped to find, we randomly selected 400 genes from the CGC as the hidden set for evaluation. From the remaining 323 CGCs, we randomly selected 20 genes from the network as prior knowledge. The other CGC genes that were not included in the prior knowledge set or hidden set were discarded. Different hidden sets and prior knowledge sets were used for 100 iterations of the uKIN algorithm. After the first propagation, the mutational frequency of each gene was used as the input for the second propagation. We utilized somatic mutation data of 24 cancer types used in the uKIN study as new information inputs of uKIN obtained from Tokheim et al. [78], who collected 1,225,917 somatic mutations in 8657 samples of TCGA somatic mutation calls (v0.2.8, https://synapse.org/MC3) [79]. We calculated the mutational frequency of a gene by dividing the frequency of somatic missense and nonsense mutations found in tumor samples by the length of the protein. To evaluate the performance of driver gene prediction, we calculated the AUROC of the top 100 scored genes resulting from the guided propagation. To compute the AUROC, only the CGC genes included in the hidden set were regarded as positive, and the other genes in the network were considered negative. Finally, we calculated the mean value from the AUROC of the 100 iterations.

### Network propagation with driver genes of each cancer type

To prioritize genes in the network according to their distance from the driver gene, we conducted network propagation using the page-rank algorithm from the NetworkX Python module. Among the 19 cancer types, we conducted network propagation for 17 cancer types, where the co-essentiality network had the highest modularity. For each cancer type, we assigned 1 for driver genes and 0 for other genes in the network as input for the personalization parameter in the page-rank algorithm. All other page-rank algorithm parameters were adjusted to their default values (damping factor = 0.85).

### Patient stratification and survival analysis

To identify driver modules capable of differentiating patient survival, we identified biological pathways located proximal to driver genes using network propagation scores of the genes in a network. To calculate the propagation scores of the genes included in each pathway, we conducted a GSEA using the GSEApy Python module. Through GSEA on network propagation score, we selected pathways significantly enriched in genes with high network propagation scores using a FDR < 0.001 and a NES > 0 as driver modules. On the other hand, the “without-network” control method used only the driver genes of each cancer type as a driver module.

The transcriptome and clinical data of patients used in this study were downloaded using the TCGAbiolinks R package [80]. For the pre-processing of gene expression data of TCGA patients, we computed the gene expression levels using read counts, which were normalized by gene length-corrected trimmed mean of M-values [81] calculated using the edgeR [82] R package. For statistical significance, we selected cancer types that contained at least 100 samples with survival data among 17 cancer types, where the co-essentiality network had the highest modularity of the driver genes. This resulted in 16 cancer types, including 7259 tumor samples.

We stratified the patients from TCGA into two groups according to their gene expression levels in the driver modules. We conducted single-sample gene set enrichment analysis (ssGSEA) on the gene expression of the selected driver modules for each patient. According to the NES of each patient, we defined the top 50% of patients as the upregulated group and the bottom 50% of patients as the downregulated group. To determine whether a survival difference existed between the upregulated and downregulated groups, we used the log-rank test and determined statistical significance. To compare the maximum capability of patient subtyping across the networks, the driver module from each network whose patient grouping had the lowest *P* value from the log-rank test was selected, and its *P* value is shown in Figure 2I. We used the Benjamini-Hochberg (BH) method to correct for multiple testing when analyzing *P* values from the log-rank test across cancer types.

### Drug–gene association data

We obtained drug–gene association data from PanDrugs [53], which assembled 18 resources with data curated by experts, and drug–gene associations collected from experimental drug screenings. The source data for PanDrugs were provided by the original authors. We used two drug–gene association types, “direct target” and “biomarker”, as drug targets. Of the 9090 drugs listed in PanDrugs, only those with a PubChem ID were selected to prevent duplicates caused by synonym usage. Finally, we considered 35,486 drug–gene associations across 7015 drugs (Table S12).

### Performance of prioritizing approved anticancer DAGs

To determine whether network propagation results can prioritize the DAGs of FDA-approved drugs in each cancer type, we performed GSEA on the propagation results of driver genes from the query cancer type using a DAG list of approved drugs as the gene set. We conducted a GSEA using the GSEApy Python module. We collected FDA-approved drugs across 16 cancer types from the TCGA(https://www.cancer.gov/about-cancer/treatment/drugs) and PanDrugs databases. The synonyms of drugs between TCGA and drug-gene association data were mapped with PubChem ID using the Pubchempy Python module [83]. Since no FDA-approved drugs of ESCA are mapped to drug-gene association data, we excluded ESCA from this analysis. Finally, 176 FDA-approved drugs across the 16 cancer types were selected for the analysis. For each cancer type, a list of genes associated with approved drugs was used for GSEA. The drug annotations in TCGA and Pandrugs were mapped by PubChem ID. The performance of the network in prioritizing approved anticancer DAGs was measured using the NES value of the GSEA results. The FDA-approved anticancer drugs and their DAGs used in this analysis are listed in Table S3.

For the “without-network” control method, we conducted GSEA using the average gene essentiality value of cell lines within the same cancer type.

### Calculation of therapeutic candidate score

For 17 cancer types, we assigned a TC score for each drug. The TC score is aggregated from the network propagation value of the DAGs by considering their RMS as follows:


$$S_{\left(d,c\right)}\;=\;\sqrt{\frac{\sum {P_{\mathrm{DAG}}}^{2}}{n_{\mathrm{DAG}}}}$$

where $S_{\left(d,c\right)}$ is the TC score of the drug *d* in cancer type *c*, $P_{\mathrm{DAG}}$ is the propagation score of the DAGs in cancer type *c*, and $n_{\mathrm{DAG}}$ is the number of DAGs.

### Performance of predicting the cytotoxic effect of drugs

To obtain drug response data of human cancer cell lines, we downloaded “secondary-screen-dose-response-curve-parameters” data from the PRISM database [84], which included 1448 compounds screened against 499 cell lines. Among the 17 cancer types, we used 15 cancer types, including cancer cell lines, which have secondary screen data in PRISM. The cytotoxic effect of the drug on each cancer type was calculated by taking the median IC_50_ values of the drug in cancer cell lines belonging to the corresponding cancer.

The network performance in predicting the cytotoxic effect of drugs on cancer was measured using Spearman’s *rho* between TC score and negative log base ten of the drug’s median IC_50_ values for the cancer. For the “without-network” control method, instead of the TC score, we used the number of driver genes among DAGs of each drug as the prediction score. The synonyms of drug information from PRSIM and PanDrugs were mapped with PubChem ID using the Pubchempy Python module [83]. We applied the BH method to correct for multiple testing when analyzing *P* values from Spearman’s correlation analyses across cancer types.

### Performance of predicting reversal gene expression effect of drugs in COADREAD

To estimate the efficacy of the drug by altering the gene expression to reverse cancer-related changes, we used the L1000 dataset from CMap [52]. From the L1000 dataset, we obtained 6056 compounds with PubChem IDs and expression profiles for 978 genes directly measured in 15 cancer cell lines whose primary site was the large intestine: CL34, HCT116, HELA, HT115, HT29, LOVO, MDST8, NCIH508, NCIH716, RKO, SNU1040, SNUC5, SW480, SW620, and SW948. We initially computed the RGE effect between the differential gene expression of COADREAD (obtained via CREEDS [51], signature ID: dz552) and the compound’s transcriptional profile using Zhang’s connection score [50], that is, the absolute value of the anti-correlated, standardized ranked connection score. Since drugs have multiple transcriptional profiles for each cell line, depending on the treatment dose and time, we calculated the maximum RGE effect for each cell line and used the median RGE effect across cell lines as a representative value. Finally, the performance of the network in predicting the drug’s RGE effect was measured using Spearman’s *rho* between the TC scores and the RGE effects of drugs that exist in both PanDrugs and CMap.

### Selection of repurposing candidates

Among the 1213 drugs approved for non-cancer diseases with drug target information in PanDrugs [53], we measured the significance of the TC score by empirical *P* value calculated from a permutation test of DAGs. Similar to the approach used for normalized modularity calculation, we constructed a reference TC score distribution for each drug. This distribution corresponded to the expected TC score of 100 randomly selected groups of genes that matched both the size and degrees of the DAGs within the network (Figure S25). To assess the significance of the TC score, we calculated its *P* value based on the reference distribution derived from random DAGs. Subsequently, we applied the Benjamini-Hochberg procedure for multiple test correction to the *P* values, and the resulting adjusted *P* values were utilized to determine the significance of the TC scores.

We selected drugs with significant TC scores (adjusted *P* ≤ 0.05) as repurposing candidates. This resulted in 145 approved drugs with new therapeutic indications across the 17 cancer types (Table S6).

### Performance of prioritizing drug repurposing candidates

To validate the repurposing candidates identified through the co-essentiality network, we leveraged clinical trial information. We considered 169 drugs labeled as “CLINICAL_CANCER”, indicating their presence clinical trial records related to cancer, as a positive set for drug repurposing (Table S12). We assessed the performance of the drug repurposing by measuring the number of repurposing candidates (with adjusted *P* ≤ 0.05) that were included in the positive set, using the F1 score as our evaluation metric.

### Comparative analysis with an existing network-based drug repurposing method

To validate the performance of drug repurposing, we implemented the network proximity-based approach presented by Cheng and colleagues [16]. For each cancer type, the proximity between cancer driver genes and DAGs was calculated using the shortest path distances in the network. The proximity score *d*(*S*, *T*) between a set of cancer driver genes (*S*) and DAGs (*T*) was defined as:


$$d(S,T)=\frac{1}{\left\|T\right\|}\sum_{t\in T}\underset{s\in T}{\min}d(s,t)$$

where *d* (*s*, *t*) is the shortest path length between nodes *s* and *t*. The proximity scores were converted to Z-scores by comparing them against a reference distribution generated from 1000 randomly selected gene sets matched in size and degree to the original sets. In addition to evaluating the original Z-score threshold of Z < −0.15, we also assessed drug repurposing candidates using two additional thresholds (Z < −0.1 and Z < −0.05) to ensure a robust comparison. The performance was evaluated using drugs with cancer-related clinical trials as the gold standard. The toolbox package for the network proximity calculation can be downloaded from github.com/emreg00/toolbox. The human interactome used to measure network proximity was downloaded from the original paper [16] (Data S1).

## Code availability

The source code for reproduction of the results was developed in either Python (v2.7.13) or Python (v3.7.9) and is available at a GitHub repository (https://github.com/SBIlab/CESnet-repurposing) and Zenodo repository (https://doi.org/10.5281/zenodo.14560305). The source code has also been submitted to BioCode at the National Genomics Data Center (NGDC), China National Center for Bioinformation (CNCB) (BioCode: BT007919), which is publicly accessible at https://ngdc.cncb.ac.cn/biocode/tools/BT007919.

## Supplementary Material

qzaf070_Supplementary_Data

## Data Availability

The final 16 networks used for the analysis at this study are available in the Figshare repository (https://doi.org/10.6084/m9.figshare.29899625) and are publicly available as of the date of publication.

## CRediT author statement

**Kwanghwan Lee:** Conceptualization, Data curation, Investigation, Writing – original draft, Visualization, Investigation, Validation, Methodology, Project administration. **Donghyo Kim:** Investigation, Writing – original draft, Investigation. **Inhae Kim:** Conceptualization, Writing – original draft, Methodology. **Juhee Lee:** Investigation, Methodology. **Doyeon Ha:** Methodology, Writing – original draft. **Seongsu Lim:** Data curation, Investigation. **Eunjee Kim:** Methodology. **Sin-Hyeog Im:** Conceptualization, Supervision. **Kunyoo Shin:** Conceptualization, Supervision. **Sanguk Kim:** Conceptualization, Funding acquisition, Supervision, Project administration, Writing – original draft. All authors have read and approved the final manuscript.
