# Supplementary material for: A Co-essentiality Network of Cancer Driver Genes Better Prioritizes Anticancer Drugs
Source: Genomics Proteomics Bioinformatics. 2025 Sep 26;23(6):qzaf070. doi: 10.1093/gpbjnl/qzaf070 (PMC13221244; doi:10.1093/gpbjnl/qzaf070)
Supplement: qzaf070_Supplementary_Data [file qzaf070_supplementary_data.zip › Figure_S10.pdf]

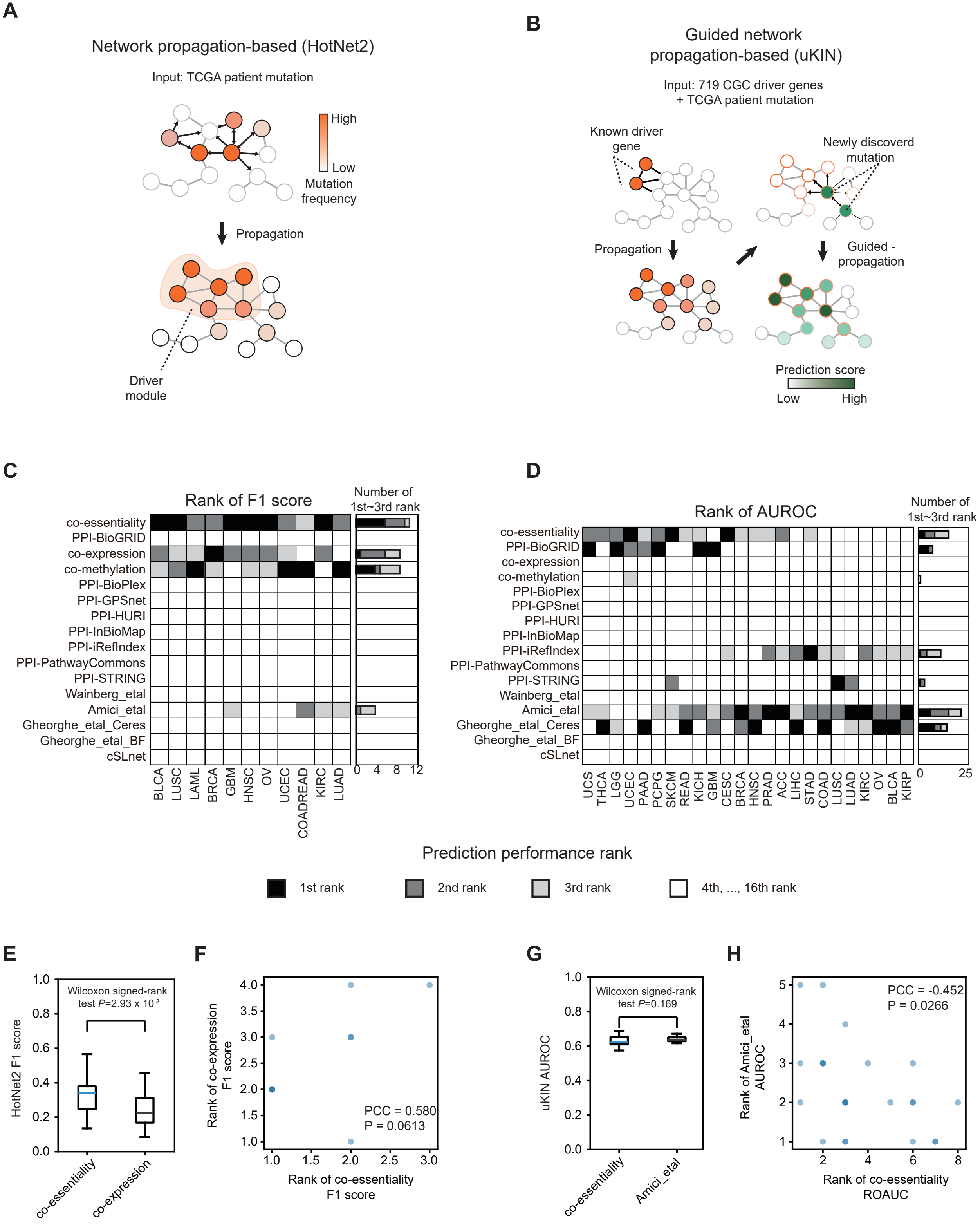
A
Network propagation-based (HotNet2)
Input: TCGA patient mutation
High
Low
Mutation frequency
Propagation
Driver module
B
Guided network propagation-based (uKIN)
Input: 719 CGC driver genes + TCGA patient mutation
Known driver gene
Newly discovered mutation
Propagation
Guided - propagation
Prediction score
Low
High
C
Rank of F1 score
Number of 1st~3rd rank
co-essentiality
PPI-BioGRID
co-expression
co-methylation
PPI-BioPlex
PPI-GPSnet
PPI-HURI
PPI-InBioMap
PPI-iRefIndex
PPI-PathwayCommons
PPI-STRING
Wainberg_etal
Amici_etal
Gheorghe_etal_Ceres
Gheorghe_etal_BF
cSLnet
BLCA
LUSC
LAML
BRCA
GBM
HNSC
OV
UCEC
COADREAD
KIRC
LUAD
0
4
8
12
D
Rank of AUROC
Number of 1st~3rd rank
co-essentiality
PPI-BioGRID
co-expression
co-methylation
PPI-BioPlex
PPI-GPSnet
PPI-HURI
PPI-InBioMap
PPI-iRefIndex
PPI-PathwayCommons
PPI-STRING
Wainberg_etal
Amici_etal
Gheorghe_etal_Ceres
Gheorghe_etal_BF
cSLnet
UCS
THCA
LGG
UCEC
PAAD
PCPG
SKCM
READ
KICH
GBM
CESC
BRCA
HNSC
PRAD
ACC
LIHC
STAD
COAD
LUSC
LUAD
KIRC
OV
BLCA
KIRP
0
25
Prediction performance rank
1st rank
2nd rank
3rd rank
4th, ..., 16th rank
E
Wilcoxon signed-rank test P=2.93 x 10-3
HotNet2 F1 score
1.0
0.8
0.6
0.4
0.2
0.0
co-essentiality
co-expression
F
Rank of co-expression F1 score
4.0
3.5
3.0
2.5
2.0
1.5
1.0
1.0
1.5
2.0
2.5
3.0
Rank of co-essentiality F1 score
PCC = 0.580
P = 0.0613
G
Wilcoxon signed-rank test P=0.169
uKIN AUROC
1.0
0.8
0.6
0.4
0.2
0.0
co-essentiality
Amici_etal
H
Rank of Amici_etal AUROC
5
4
3
2
1
2
4
6
8
Rank of co-essentiality ROAUC
PCC = -0.452
P = 0.0266
