# Supplementary material for: A Co-essentiality Network of Cancer Driver Genes Better Prioritizes Anticancer Drugs
Source: Genomics Proteomics Bioinformatics. 2025 Sep 26;23(6):qzaf070. doi: 10.1093/gpbjnl/qzaf070 (PMC13221244; doi:10.1093/gpbjnl/qzaf070)
Supplement: qzaf070_Supplementary_Data [file qzaf070_supplementary_data.zip › Figure_S16.pdf]

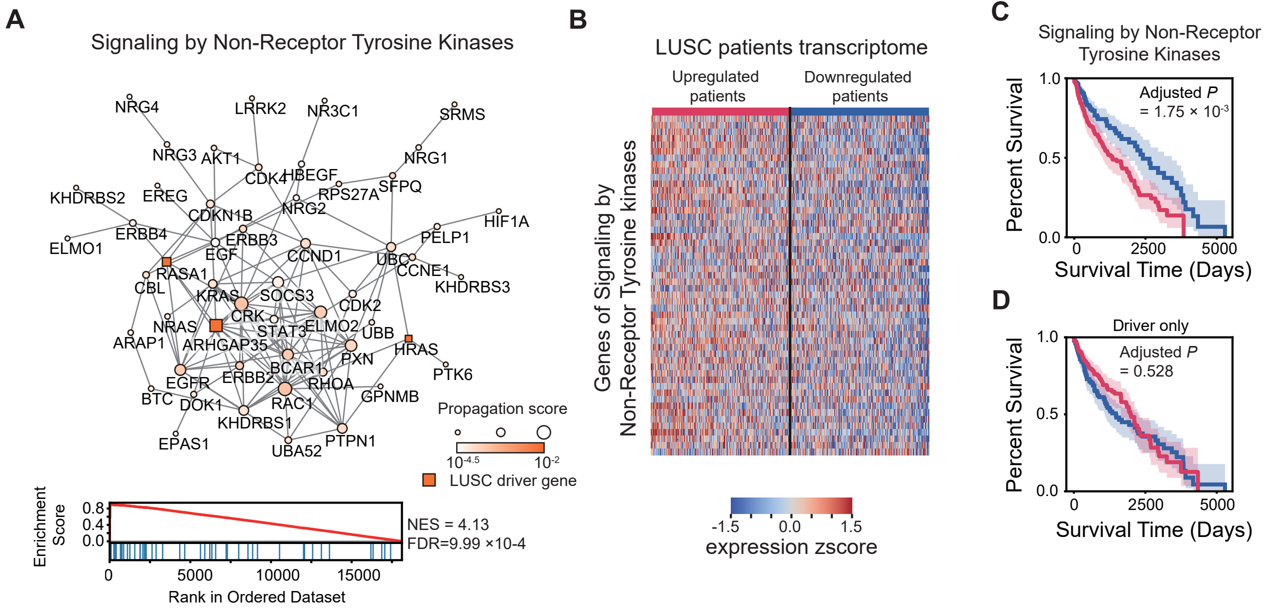
A
Signaling by Non-Receptor Tyrosine Kinases
NRG4
LRRK2
NR3C1
SRMS
NRG3
AKT1
NRG1
CDK4
HBEGF
KHDRBS2
EREG
RPS27A
SFPQ
CDKN1B
NRG2
HIF1A
ERBB4
ERBB3
PELP1
ELMO1
EGF
CCND1
UBC
RASA1
CCNE1
CBL
KHDRBS3
KRAS
SOCS3
CDK2
CRK
NRAS
STAT3
ELMO2
UBB
ARAP1
ARHGAP35
HRAS
PXN
BCAR1
PTK6
EGFR
ERBB2
RHOA
BTC
DOK1
RAC1
GPNMB
KHDRBS1
EPAS1
UBA52
PTPN1
Propagation score
10-4.5
10-2
LUSC driver gene
Enrichment Score
0.8
0.4
0.0
NES = 4.13
FDR=9.99 ×10-4
0
5000
10000
15000
Rank in Ordered Dataset
B
LUSC patients transcriptome
Upregulated patients
Downregulated patients
Genes of Signaling by Non-Receptor Tyrosine kinases
-1.5
0.0
1.5
expression zscore
C
Signaling by Non-Receptor Tyrosine Kinases
Adjusted P = 1.75 × 10-3
Percent Survival
1.0
0.5
0.0
0
2500
5000
Survival Time (Days)
D
Driver only
Adjusted P = 0.528
Percent Survival
1.0
0.5
0.0
0
2500
5000
Survival Time (Days)
