# Supplementary material for: A Co-essentiality Network of Cancer Driver Genes Better Prioritizes Anticancer Drugs
Source: Genomics Proteomics Bioinformatics. 2025 Sep 26;23(6):qzaf070. doi: 10.1093/gpbjnl/qzaf070 (PMC13221244; doi:10.1093/gpbjnl/qzaf070)
Supplement: qzaf070_Supplementary_Data [file qzaf070_supplementary_data.zip › Figure_S20.pdf]

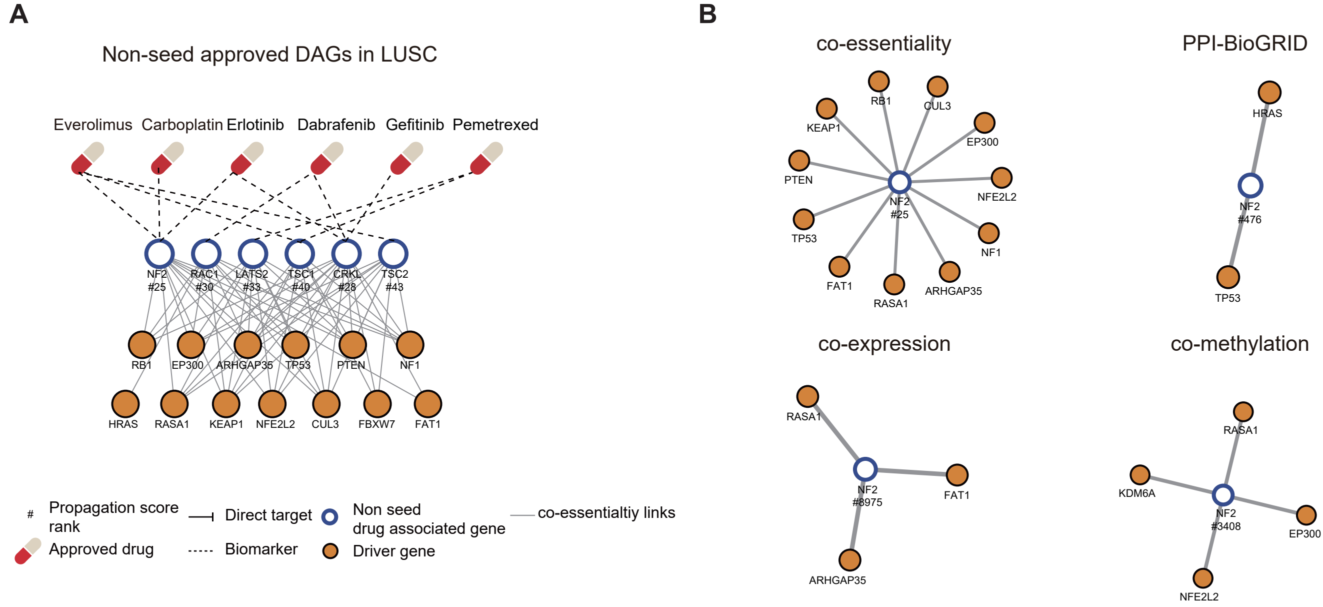
A
Non-seed approved DAGs in LUSC
Everolimus
Carboplatin
Erlotinib
Dabrafenib
Gefitinib
Pemetrexed
NF2 #25
RAC1 #30
LATS2 #33
TSC1 #40
CRKL #28
TSC2 #43
RB1
EP300
ARHGAP35
TP53
PTEN
NF1
HRAS
RASA1
KEAP1
NFE2L2
CUL3
FBXW7
FAT1
Propagation score rank
Approved drug
Direct target
Biomarker
Non seed drug associated gene
Driver gene
co-essentialtiy links
B
co-essentiality
RB1
CUL3
KEAP1
EP300
PTEN
NFE2L2
NF2 #25
TP53
NF1
FAT1
RASA1
ARHGAP35
PPI-BioGRID
HRAS
NF2 #476
TP53
co-expression
RASA1
NF2 #8975
FAT1
ARHGAP35
co-methylation
RASA1
KDM6A
NF2 #3408
EP300
NFE2L2
