# Supplementary material for: A Co-essentiality Network of Cancer Driver Genes Better Prioritizes Anticancer Drugs
Source: Genomics Proteomics Bioinformatics. 2025 Sep 26;23(6):qzaf070. doi: 10.1093/gpbjnl/qzaf070 (PMC13221244; doi:10.1093/gpbjnl/qzaf070)
Supplement: qzaf070_Supplementary_Data [file qzaf070_supplementary_data.zip › Figure_S22.pdf]

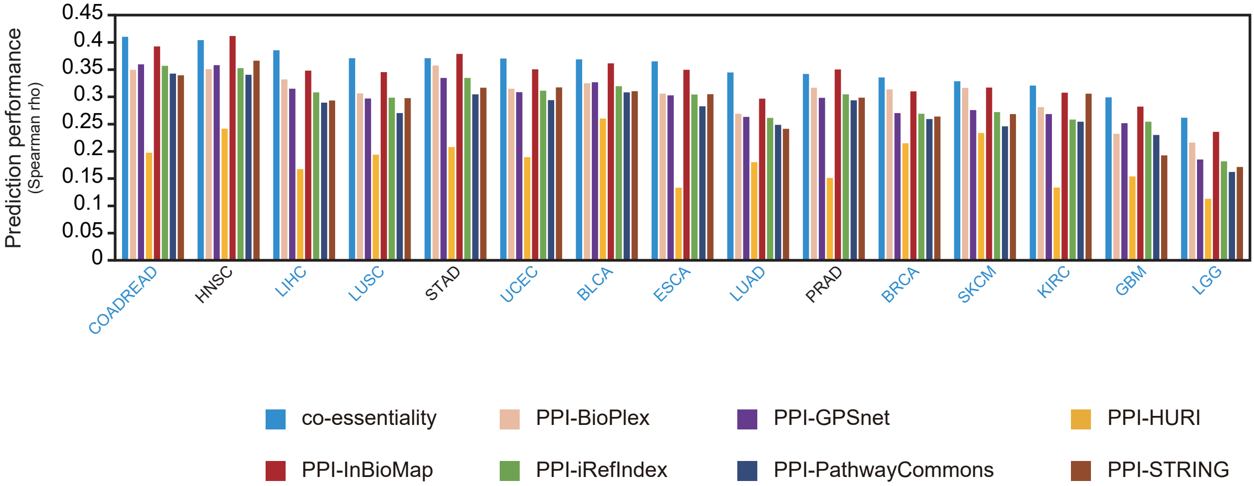

Prediction performance
(Spearman rho)
0.45
0.4
0.35
0.3
0.25
0.2
0.15
0.1
0.05
0
COADREAD
HNSC
LIHC
LUSC
STAD
UCEC
BLCA
ESCA
LUAD
PRAD
BRCA
SKCM
KIRC
GBM
LGG
co-essentiality
PPI-BioPlex
PPI-GPSnet
PPI-HURI
PPI-InBioMap
PPI-iRefIndex
PPI-PathwayCommons
PPI-STRING
