# Supplementary material for: A Co-essentiality Network of Cancer Driver Genes Better Prioritizes Anticancer Drugs
Source: Genomics Proteomics Bioinformatics. 2025 Sep 26;23(6):qzaf070. doi: 10.1093/gpbjnl/qzaf070 (PMC13221244; doi:10.1093/gpbjnl/qzaf070)
Supplement: qzaf070_Supplementary_Data [file qzaf070_supplementary_data.zip › Supplementary material captions.docx]

**Supplementary material**

**Figure S1**  **Curated gene set enrichment of co-essentiality links**

Enrichment of the co-essentiality links to six curated gene sets: CORUM, KEGG, Reactome, GO:BP, GO:MF, and GO:CC. Co-essentiality links were ranked and binned (*n* = 10,000). The enrichment of the co-essentiality links in each bin is indicated in blue dots. The expected enrichment of co-essentiality links in each bin is indicated by gray dots.

**Figure S2 Comparative analyses of cancer-related pathways across molecular networks using gene set enrichment analysis and relative modularity distributions**

**A.** GSEA, Gene set enrichment analysis of CRPs, cancer-related pathways ranked by relative modularity in each network. A barplot shows the NES with corresponding significance for the co-essentiality, PPI-BioGRID, co-expression, and co-methylation networks(**, FDR < 0.01; ns, not significant). **B.** Boxplot distribution of relative modularity values for the 31 CRPs across the four networks. Statistical comparisons were performed using the Mann-Whitney U test with the co-essentiality network as the reference (**, *P* < 0.01, ns = not significant).

**Figure S3 Empirical distribution of modularity of the degree controlled random nodes for LUSC driver genes**

The x-axis showed the value of cohesiveness used for the modularity measure, while the y-axis denotes the fraction of the number of random modules against 100 permutations. The red pointers are observed cohesiveness value from each network. The value *m* in the upper right corner is the normalized modularity value calculated from the empirical distribution of the cohesiveness values.

**Figure S4 Modularity of cancer drivers from cancer gene census in the four networks**

The modularity calculated from the subnetwork of driver genes from CGC, cancer gene census for the four networks: co-essentiality, PPI-BioGRID, co-expression, and co-methylation. For blue-colored cancer types, the co-essentiality network showed the highest modularity among the four networks.

**Figure S5 Scaled clustering coefficient of cancer driver genes in the four networks**

Scaled clustering coefficient calculated from the subnetwork of driver genes across 19 TCGA cancer types for the four networks: co-essentiality, PPI-BioGRID, co-expression, and co-methylation. For blue-colored cancer types, the co-essentiality network showed the highest scaled clustering coefficient among the four networks.

**Figure S6 Correlation between the modularity of driver genes in the co-essentiality network and the number of cell lines used to construct the co-essentiality network**

The purple, blue, and green colors represent co-essentiality networks constructed with 100, 300, and 500 randomly selected cell lines, respectively. The sky-blue lines indicate the co-essentiality network constructed using all 769 cell lines in the DepMap 20q2 dataset.

**Figure S7 Performance of the co-essentiality network for cancer driver gene identification compared with that of seven different protein-protein interaction networks**

Performance of driver gene identification for eight networks: co-essentiality, BioPlex, GPSnet, HURI, Inbiomap, iRefIndex, Pathway Commons, and STRING. For blue-colored cancer types, the co-essentiality network showed the best performance among the eight networks.

**Figure S8 Performance of the co-essentiality network for cancer driver gene identification compared with that of other co-essentiality networks and the genetic interaction network**

Performance of driver gene identification for other co-essentiality networks (Wainberg_etal, Amici_etal, Gheorghe_etal_Ceres, and Gheorghe_etal_BF), and the genetic interaction network based on synthetic-lethal relationship (cSLnet). For blue-colored cancer types, the co-essentiality network showed the best performance among the three networks.

**Figure S9 Correlation between performance of the co-essentiality network for cancer driver gene identification and frequency of cancer types used for co-essentiality network construction**

The correlation was calculated by Spearman’s correlation coefficient R.

**Figure S10 Performance for Hotnet2-based and uKIN-based driver gene identification**

Schematic overviews of the HotNet2 (**A**) and uKIN (**B**) algorithms. Heatmaps showing performance ranks of driver gene identification across 16 networks, using HotNet2 F1 scores (**C**) and uKIN AUROC (**D**). Black, dark gray, and light gray indicate 1st, 2nd, and 3rd place, respectively; white indicates ranks below 4th. Boxplots comparing the top two performing networks for HotNet2 F1 scores (**E**: co-essentiality *vs*. co-expression) and uKIN AUROC (**G**: co-essentiality vs. Amici_etal). Significance was assessed by the Wilcoxon signed-rank test**.** Scatter plots comparing performance ranks across cancer types for HotNet2 F1 scores (**F**: 11 cancer types; x-axis: the co-essentiality network, y-axis: the co-expression network) and uKIN AUROC (**H**: 24 cancer types; x-axis: the co-essentiality network, y-axis: Amici_etal). Pearson’s correlation coefficient was used to measure correlation between the two network’s ranks.

**Figure S11 Survival plot of patient subgroups in 16 cancer types stratified by the co-essentiality network**

Red lines represent patient groups with upregulated driver module expression. Blue lines represent patient groups with downregulated driver module expression. The significance of survival difference between patient groups is shown as the *P* of the log-rank test. Cancer types denoted with a blank box had no significant driver modules in the network. Benjamini-Hochberg method was applied to correct for multiple testing when analyzing p-values from log-rank test across cancer types.

**Figure S12 Survival plot of patient subgroups in 16 cancer types stratified by the PPI-BioGRID**

Red lines represent patient groups with upregulated driver module expression. Blue lines represent patient groups with downregulated driver module expression. The significance of survival difference between patient groups is shown as the *P* of the log-rank test. Cancer types denoted with a blank box had no significant driver modules in the network. Benjamini-Hochberg method was applied to correct for multiple testing when analyzing p values from log-rank test across cancer types.

**Figure S13 Survival plot of patient subgroups in 16 cancer types stratified by the co-expression network**

Red lines represent patient groups with upregulated driver module expression. Blue lines represent patient groups with downregulated driver module expression. The significance of survival difference between patient groups is shown as the *P* of the log-rank test. Cancer types denoted with a blank box had no significant driver modules in the network. Benjamini-Hochberg method was applied to correct for multiple testing when analyzing p values from log-rank test across cancer types.

**Figure S14 Survival plot of patient subgroups in 16 cancer types stratified by the co-methylation network**

Red lines represent patient groups with upregulated driver module expression. Blue lines represent patient groups with downregulated driver module expression. The significance of survival difference between patient groups is shown as the *P* of the log-rank test. Cancer types denoted with a blank box had no significant driver modules in the network. Benjamini-Hochberg method was applied to correct for multiple testing when analyzing p-values from log-rank test across cancer types.

**Figure S15 Survival plot of patient subgroups in 16 cancer types stratified by driver genes of each cancer type**

Red lines represent patient groups with upregulated driver gene expression. Blue lines represent patient groups with downregulated driver gene expression. The significance of survival difference between patient groups is shown as the *P* of the log-rank test. Benjamini-Hochberg method was applied to correct for multiple testing when analyzing Ps from log-rank test across cancer types.

**Figure S16 Patient stratification using the co-essentiality network module (Signaling by Non-Receptor Tyrosine Kinases pathway) in lung squamous cell carcinoma**

**A.** Subnetwork of the co-essentiality network for signaling by nonreceptor tyrosine kinase pathway (up). Enrichment plot of the co-essentiality network resulting from GSEA on Signaling by Non-Receptor tyrosine Kinase pathway (bottom). **B.** Heatmap of LUSC, lung squamous cell carcinoma gene expression in Signaling by Non-Receptor Tyrosine Kinases pathway. **C.** Survival plot of LUSC patient subgroups stratified by gene expression of signaling by the nonreceptor tyrosine kinase pathway. **D.** Survival plot of LUSC patient subgroups stratified by LUSC driver genes. Red and blue lines represent the upregulated and downregulated patient groups, respectively. Benjamini-Hochberg method was applied to correct for multiple testing when analyzing p-values from log-rank test across cancer types.

**Figure S17 Performance of the co-essentiality network for approved anticancer drug-associated gene prioritization compared with that of seven PPI networks**

**A.** The performance for anticancer DAG, drug-associated gene prioritization was measured using the NES for eight networks: co-essentiality, BioPlex, GPSnet, HURI, Inbiomap, iRefIndex, Pathway Commons, and STRING. **B.** The prioritization performance for targets of approved anticancer drug was measured using the NES. **C.** The prioritization performance for biomarkers of approved anticancer drug was measured using the NES. For blue-colored cancer types, the co-essentiality network showed the highest NES among the eight networks.

**Figure S18 Performance of the co-essentiality network for approved anticancer drug-associated gene prioritization compared with that of other co-essentiality networks and a genetic interaction network**

**A.** The performance for anticancer DAG, drug-associated gene prioritization was measured using the NES for other co-essentiality networks (Wainberg_etal, Amici_etal, Gheorghe_etal_Ceres, and Gheorghe_etal_BF), and the genetic interaction network(cSLnet). **B.** The prioritization performance for targets of approved anticancer drug was measured using the NES. **C.** The prioritization performance for biomarkers of approved anticancer drug was measured using the NES. For blue-colored cancer types, the co-essentiality network showed the highest NES among the six networks.

**Figure S19 Performance of the co-essentiality network for targets and biomarkers of approved anticancer drugs prioritization compared with that of the four networks**

**A.** The prioritization performance for targets of approved anticancer drug was measured using the NES for four networks: co-essentiality, BioGRID, co-expression, co-methylation. **B.** The prioritization performance for biomarkers of approved anticancer drug was measured using the NES. For blue-colored cancer types, the co-essentiality network showed the highest NES among the four networks.

**Figure S20 Prioritization of anticancer drug-associated genes in Lung squamous cell carcinoma using the co-essentiality network**

**A.** Subnetwork of six approved DAGs (*NF2*, *RAC1*, *LATS2*, *TSC1*, *CRKL,* and *TSC2*) in LUSC and driver genes in their first neighbors in the co-essentiality network. Only drug-gene associations of these six genes are shown in this figure. **B.** First neighbors of *NF2* in the four networks: co-essentiality, PPI-BioGRID, co-expression, and co-methylation.

**Figure S21 Predicting drug response in cancer cells of Lung squamous cell carcinoma using therapeutic candidate score of the co-essentiality network**

**A.** Scatter plot of drug response and TC score of LUSC in the co-essentiality network and data point of candidate drug: Everolimus (red dot). **B.** A subnetwork of 36 DAGs of Everolimus and LUSC driver genes which are connected to them in the co-essentiality network.

**Figure S22 Performance of the co-essentiality network for drug response prediction compared with that of seven different protein-protein interaction networks**

The performance of drug response prediction was measured using the Spearman’s correlation coefficient (Spearman’s rho) for eight networks: co-essentiality, BioPlex, GPSnet, HURI, Inbiomap, iRefIndex, Pathway Commons, and STRING. For blue-colored cancer types, the co-essentiality network showed the best performance among the eight networks.

**Figure S23 Performance of the co-essentiality network for drug response prediction compared with that of other co-essentiality networks and genetic interaction network**

The performance of drug response prediction was measured using the Spearman’s correlation coefficient (Spearman’s rho) for other co-essentiality networks (Wainberg_etal, Amici_etal, Gheorghe_etal_Ceres, and Gheorghe_etal_BF), and the genetic interaction network (cSLnet). For blue-colored cancer types, the co-essentiality network showed the best performance among the six networks.

**Figure S24 Comparing the performance of drug response prediction using both target and biomarker information versus using only target information**

Blue colored bars are prediction performance of drug response using the TC score based on DAGs including both targets and biomarkers. Black colored bars are prediction performance of drug response using the TC score based on only target information.

**Figure S25 Empirical distribution of the therapeutic candidate score of the degree controlled random nodes for drug-associated genes of Pioglitazone hydrochloride**

The x-axis shows the value of the TC score, while the y-axis denotes the fraction of the number of random DAGs against 100 permutations. The red dot line is the observed TC score of Pioglitazone hydrochloride.

**Figure S26 Performance of the co-essentiality network for repurposing of non-cancer approved drugs compared with that of seven different protein-protein interaction networks**

The performance for repurposing of non-cancer-approved drugs was measured using the F1 score for eight networks: co-essentiality, BioPlex, GPSnet, HURI, Inbiomap, iRefIndex, Pathway Commons, and STRING. For blue-colored cancer types, the co-essentiality network showed the highest F1 score among the eight networks.

**Figure S27 Performance of the co-essentiality network for repurposing of non-cancer approved drugs compared with that of other co-essentiality networks and a genetic interaction network**

The performance for repurposing of non-cancer approved drugs was measured using the F1 score for other co-essentiality network (Wainberg_etal, Amici_etal, Gheorghe_etal_Ceres, and Gheorghe_etal_BF), and a genetic interaction network (cSLnet). For blue-colored cancer types, the co-essentiality network showed the highest F1 score among the three networks.

**Figure S28 Comparison of *in silico* drug repurposing performance between co-essentiality network method and Cheng et al. method**

Drug repurposing performance was evaluated using clinical trial records as validation. Prediction performance was measured using the F1 score. Yellow spectrum bars indicate the performance of the Cheng et al. method at three different network proximity thresholds z for identifying repurposed drugs. For cancer types shown in blue, the co-essentiality network demonstrated superior performance compared to all three alternative approaches.

**F****igure S29 Performance comparison of cancer driver gene identification across three correlation-based networks (co-essentiality, co-expression, and co-methylation) using different network link thresholds ranging from 0.0 to 5.0**

Red bars indicate results at the selected threshold t > 2.0 used in this study.

**Table S1 Relative modularity of 186 KEGG pathways on four networks and the cancer-related pathway list**

**Table S2 Information of the 16 networks used in this study**

**Table S3 List of FDA approved anticancer drugs and their corresponding drug associated genes**

**Table S4 FDA-approved drug-associated genes of skin cutaneous melanoma among the top 50 genes with the highest propagation values in the co-essentiality network**

**Table S5 FDA-approved drug-associated genes of lung squamous cell carcinoma among the top 50 genes with the highest propagation values in the co-essentiality network**

**Table S6 List of drug repurposing candidates predicted by the co-essentiality network**

**Table S7** **Three repurposing candidates in lung adenocarcinoma predicted by the co-essentiality network but not by other networks**

**Table S8 AUROC of driver gene identification of three correlation-based networks according to six threshold values (t)**

**Table S9**  **Comparison of AUROC for driver gene identification using the correlation-based and mutual information-based co-expression networks across six threshold values (t)**

**Table S10 The contingency tables of the four networks to cancer-related pathway enrichment**

**Table S11 “Tier 1” driver genes in the cancer gene census and matched TCGA cancer types**

**Table S12 Drug-gene association and drug information**
