# Supplementary material for: A Co-essentiality Network of Cancer Driver Genes Better Prioritizes Anticancer Drugs
Source: Genomics Proteomics Bioinformatics. 2025 Sep 26;23(6):qzaf070. doi: 10.1093/gpbjnl/qzaf070 (PMC13221244; doi:10.1093/gpbjnl/qzaf070)
Supplement: qzaf070_Supplementary_Data [file qzaf070_supplementary_data.zip › Table S2.docx]

**Table S2 Information of the 16 networks used in this study**

| Network | Node  number | Link  number | Essential gene  number | Essential gene  fraction | Rank of  fraction |
| --- | --- | --- | --- | --- | --- |
| Co-essentiality | 18,119 | 8,105,180 | 2070 | 11.4% | 9 |
| PPI-BioGRID | 18,708 | 434,527 | 2011 | 10.7% | 13 |
| Co-expression | 19,120 | 12,403,225 | 2120 | 11.1% | 11 |
| Co-methylation | 16,333 | 10,193,089 | 1732 | 10.6% | 14 |
| PPI-BioPlex | 12,848 | 86,968 | 1700 | 13.2% | 3 |
| PPI-GPSnet | 15,124 | 167,854 | 1948 | 12.9% | 5 |
| PPI-HURI | 8124 | 51,816 | 1009 | 12.4% | 6 |
| PPI-Inbiomap | 17,421 | 608,161 | 2014 | 11.6% | 7 |
| PPI-iRefIndex | 14,955 | 152,147 | 1930 | 12.9% | 4 |
| PPI-PathwayCommons | 17,646 | 88,329 | 2035 | 11.5% | 8 |
| PPI-STRING | 18,119 | 770,875 | 2070 | 11.4% | 9 |
| Wainberg_etal | 6351 | 14,980 | 1191 | 18.8% | 1 |
| Amici_etal | 19,082 | 1,040,194 | 2089 | 10.9% | 12 |
| Gheorghe_etal_Ceres | 12,910 | 359,564 | 1847 | 14.3% | 2 |
| Gheorghe_etal_BF | 2025 | 6,244 | 210 | 10.4% | 15 |
| cSLnet | 8368 | 21,450 | 741 | 8.9% | 16 |
