# Supplementary material for: A Co-essentiality Network of Cancer Driver Genes Better Prioritizes Anticancer Drugs
Source: Genomics Proteomics Bioinformatics. 2025 Sep 26;23(6):qzaf070. doi: 10.1093/gpbjnl/qzaf070 (PMC13221244; doi:10.1093/gpbjnl/qzaf070)
Supplement: qzaf070_Supplementary_Data [file qzaf070_supplementary_data.zip › Table S4.docx]

**Table S4 FDA-approved drug-associated genes of skin cutaneous melanoma among the top 50 genes with the highest propagation values in the co-essentiality network**

| Rank | Gene | Driver | Direct Target | Biomarker |
| --- | --- | --- | --- | --- |
| 1 | TP53 | O |  | VEMURAFENIB, PACLITAXEL, DABRAFENIB, VORINOSTAT, 5-FLUOROURACIL, TRAMETINIB |
| 2 | BRAF | O | DABRAFENIB, VEMURAFENIB | PACLITAXEL, VORINOSTAT, 5-FLUOROURACIL, TRAMETINIB, COBIMETINIB, DABRAFENIB MESYLATE |
| 3 | RAC1 | O |  | DABRAFENIB, VEMURAFENIB |
| 5 | CTNNB1 | O |  | TRAMETINIB |
| 7 | KRAS | O |  | VEMURAFENIB, PACLITAXEL, DABRAFENIB, 5-FLUOROURACIL, TRAMETINIB, COBIMETINIB |
| 8 | RB1 | O |  | VORINOSTAT, TRAMETINIB |
| 10 | PTEN | O |  | PACLITAXEL, VORINOSTAT, VEMURAFENIB |
| 11 | NF1 | O |  | DABRAFENIB, COBIMETINIB, VEMURAFENIB, TRAMETINIB |
| 12 | MAP2K1 | O | COBIMETINIB, TRAMETINIB | DABRAFENIB MESYLATE, DABRAFENIB, VEMURAFENIB, COBIMETINIB FUMARATE, 1187431-43-1 |
| 14 | NRAS | O |  | DABRAFENIB, COBIMETINIB, VEMURAFENIB, 5-FLUOROURACIL, TRAMETINIB |
| 18 | HRAS | O |  | TRAMETINIB |
| 20 | KIT | O |  | TRAMETINIB |
| 22 | GNA11 | O |  | VORINOSTAT, TRAMETINIB |
| 23 | CDKN2A | O |  | PACLITAXEL, TRAMETINIB |
| 27 | CDKN1A | X |  | PACLITAXEL, 5-FLUOROURACIL |
| 30 | ATM | X |  | PACLITAXEL, TRAMETINIB |
| 32 | CRKL | X |  | DABRAFENIB, VEMURAFENIB |
| 34 | SOX10 | X |  | VEMURAFENIB |
| 40 | SOX9 | X |  | 5-FLUOROURACIL |
| 47 | RAF1 | X | DABRAFENIB | VEMURAFENIB |
