# Supplementary material for: A Co-essentiality Network of Cancer Driver Genes Better Prioritizes Anticancer Drugs
Source: Genomics Proteomics Bioinformatics. 2025 Sep 26;23(6):qzaf070. doi: 10.1093/gpbjnl/qzaf070 (PMC13221244; doi:10.1093/gpbjnl/qzaf070)
Supplement: qzaf070_Supplementary_Data [file qzaf070_supplementary_data.zip › Table S5.docx]

**Table S5 FDA-approved drug-associated genes of lung squamous cell carcinoma among the top 50 genes with the highest propagation values in the co-essentiality network**

| Rank | Gene | Driver | Direct Target | Biomarker |
| --- | --- | --- | --- | --- |
| 2 | TP53 | O |  | GEMCITABINE, PACLITAXEL, DABRAFENIB, TRAMETINIB, CARBOPLATIN, TOPOTECAN, LORLATINIB, CRIZOTINIB, DOCETAXEL, ERLOTINIB |
| 6 | PTEN | O |  | EVEROLIMUS, GEMCITABINE, PACLITAXEL, GEFITINIB, CARBOPLATIN, TOPOTECAN, DACOMITINIB, PEMETREXED, ERLOTINIB |
| 7 | EP300 | O |  | PEMETREXED |
| 8 | NF1 | O |  | DABRAFENIB, ERLOTINIB, EVEROLIMUS, TRAMETINIB |
| 10 | KDM6A | O |  | PEMETREXED |
| 12 | FBXW7 | O |  | DOCETAXEL |
| 14 | PIK3CA | O |  | EVEROLIMUS, GEMCITABINE, PACLITAXEL, DABRAFENIB, TRAMETINIB, CARBOPLATIN, TOPOTECAN, PEMETREXED, DOCETAXEL |
| 15 | RB1 | O |  | METHOTREXATE, TOPOTECAN, LORLATINIB, EVEROLIMUS, TRAMETINIB |
| 17 | HLA-A | O |  | GEFITINIB |
| 19 | FGFR2 | O |  | GEFITINIB, TRAMETINIB |
| 20 | NOTCH1 | O |  | METHOTREXATE, PACLITAXEL, DOCETAXEL, EVEROLIMUS |
| 21 | HRAS | O |  | TRAMETINIB, DOCETAXEL, EVEROLIMUS, GEMCITABINE |
| 22 | CDKN2A | O |  | GEMCITABINE, PACLITAXEL, TRAMETINIB, CARBOPLATIN, CRIZOTINIB, ALECTINIB |
| 25 | NF2 | X |  | EVEROLIMUS, CARBOPLATIN, ERLOTINIB |
| 28 | CRKL | X |  | DABRAFENIB, GEFITINIB, ERLOTINIB |
| 30 | RAC1 | X |  | DABRAFENIB |
| 33 | LATS2 | X |  | PEMETREXED |
| 40 | TSC2 | X |  | EVEROLIMUS |
| 43 | TSC1 | X |  | PEMETREXED, EVEROLIMUS |
