# Supplementary material for: A Co-essentiality Network of Cancer Driver Genes Better Prioritizes Anticancer Drugs
Source: Genomics Proteomics Bioinformatics. 2025 Sep 26;23(6):qzaf070. doi: 10.1093/gpbjnl/qzaf070 (PMC13221244; doi:10.1093/gpbjnl/qzaf070)
Supplement: qzaf070_Supplementary_Data [file qzaf070_supplementary_data.zip › Table S7.docx]

**Table S7** **Three repurposing candidates in LUAD predicted by the co-essentiality network but not by other networks**

| Drug | TC score | Adjusted P | Clinical trial records in cancer |
| --- | --- | --- | --- |
| ATOVAQUONE | $7.81\times{10}^{-5}$ | $2.57\times{10}^{-3}$ | O |
| TERIFLUNOMIDE | $7.42\times{10}^{-5}$ | $1.09\times{10}^{-2}$ | X |
| EFLORNITHINE | $6.73\times{10}^{-5}$ | $1.77\times{10}^{-2}$ | O |
