# Supplementary material for: A Co-essentiality Network of Cancer Driver Genes Better Prioritizes Anticancer Drugs
Source: Genomics Proteomics Bioinformatics. 2025 Sep 26;23(6):qzaf070. doi: 10.1093/gpbjnl/qzaf070 (PMC13221244; doi:10.1093/gpbjnl/qzaf070)
Supplement: qzaf070_Supplementary_Data [file qzaf070_supplementary_data.zip › Table S8.docx]

**Table S8 AUROC of driver gene identification of three correlation-based networks according to six threshold values(t)**

| Threshold t | co-essentiality | co-expression | co-methylation |
| --- | --- | --- | --- |
| 0 | 0.815 | 0.737 | 0.583 |
| 1 | 0.823 | 0.747 | 0.583 |
| 2 | 0.814 | 0.758 | 0.583 |
| 3 | 0.773 | 0.681 | 0.556 |
| 4 | 0.711 | 0.539 | 0.516 |
| 5 | 0.662 | 0.516 | 0.502 |
