# Supplementary material for: A Co-essentiality Network of Cancer Driver Genes Better Prioritizes Anticancer Drugs
Source: Genomics Proteomics Bioinformatics. 2025 Sep 26;23(6):qzaf070. doi: 10.1093/gpbjnl/qzaf070 (PMC13221244; doi:10.1093/gpbjnl/qzaf070)
Supplement: qzaf070_Supplementary_Data [file qzaf070_supplementary_data.zip › Table S9.docx]

**Table S9**  **Comparison of AUROC for driver gene identification using the correlation-based and mutual information-based co-expression networks across six threshold values (t)**

| Threshold t | Pearson correlation coefficient  (PCC) | Maximal information coefficient  (MIC) |
| --- | --- | --- |
| 0 | 0.737 | 0.611 |
| 1 | 0.747 | 0.648 |
| 2 | 0.758 | 0.721 |
| 3 | 0.681 | 0.698 |
| 4 | 0.539 | 0.635 |
| 5 | 0.516 | 0.580 |
