# Supplementary material for: A Co-essentiality Network of Cancer Driver Genes Better Prioritizes Anticancer Drugs
Source: Genomics Proteomics Bioinformatics. 2025 Sep 26;23(6):qzaf070. doi: 10.1093/gpbjnl/qzaf070 (PMC13221244; doi:10.1093/gpbjnl/qzaf070)
Supplement: qzaf070_Supplementary_Data [file qzaf070_supplementary_data.zip › Figure_S7.pdf]

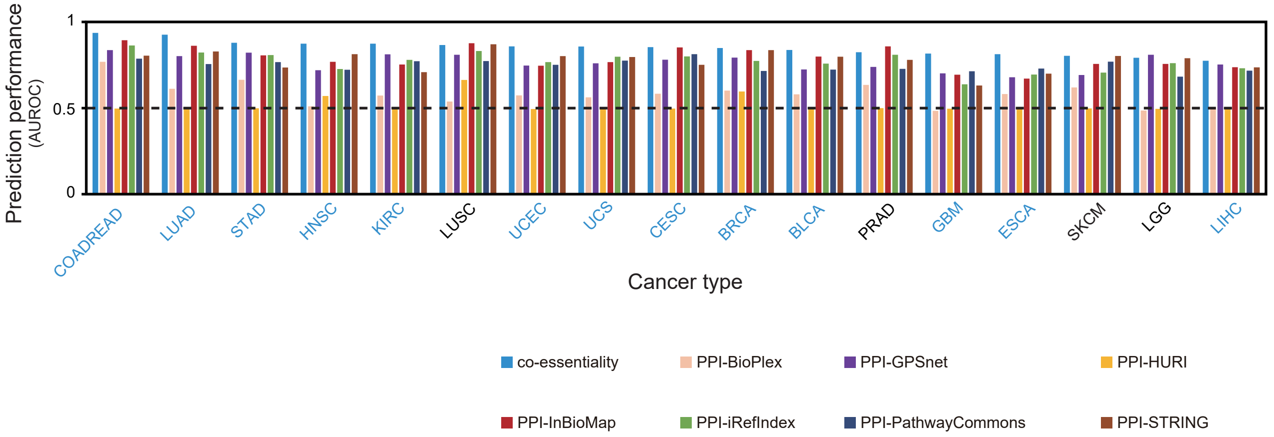

Prediction performance
(AUROC)
1
0.5
0
COADREAD
LUAD
STAD
HNSC
KIRC
LUSC
UCEC
UCS
CESC
BRCA
BLCA
PRAD
GBM
ESCA
SKCM
LGG
LIHC
Cancer type
co-essentiality
PPI-BioPlex
PPI-GPSnet
PPI-HURI
PPI-InBioMap
PPI-iRefIndex
PPI-PathwayCommons
PPI-STRING
