# Supplementary figures and images for: A Co-essentiality Network of Cancer Driver Genes Better Prioritizes Anticancer Drugs
Source: Genomics Proteomics Bioinformatics. 2025 Sep 26;23(6):qzaf070. doi: 10.1093/gpbjnl/qzaf070 (PMC13221244; doi:10.1093/gpbjnl/qzaf070)

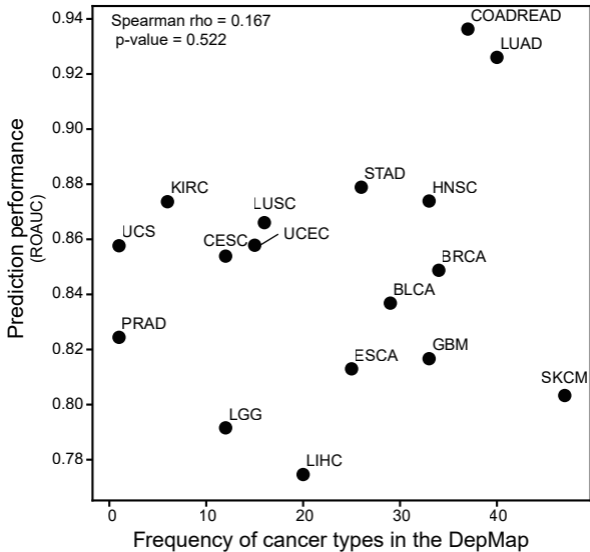

Supplement: qzaf070_Supplementary_Data [file qzaf070_supplementary_data.zip › Figure_S9.pdf]

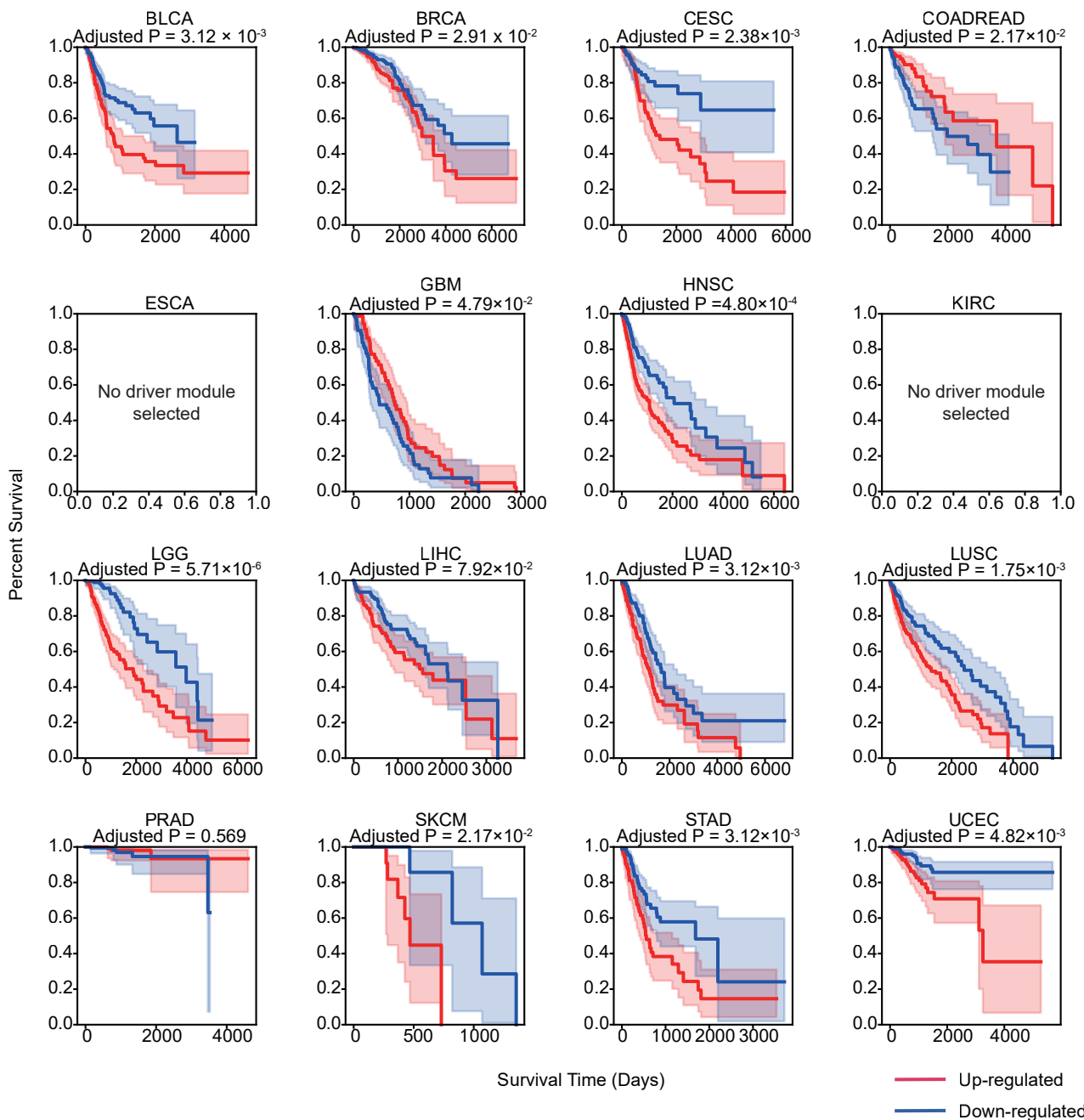

Supplement: qzaf070_Supplementary_Data [file qzaf070_supplementary_data.zip › Figure_S11.pdf]

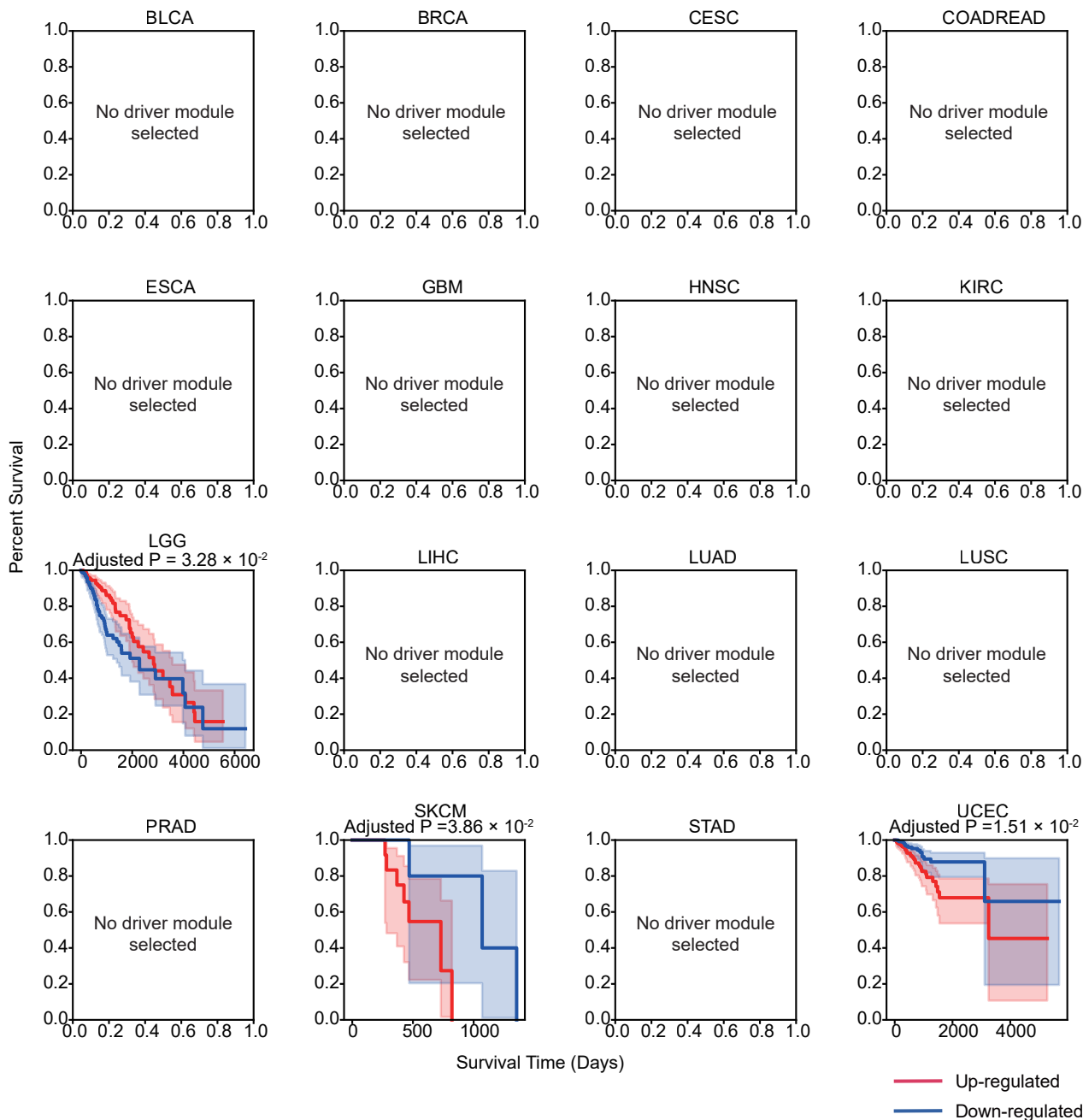

Supplement: qzaf070_Supplementary_Data [file qzaf070_supplementary_data.zip › Figure_S12.pdf]

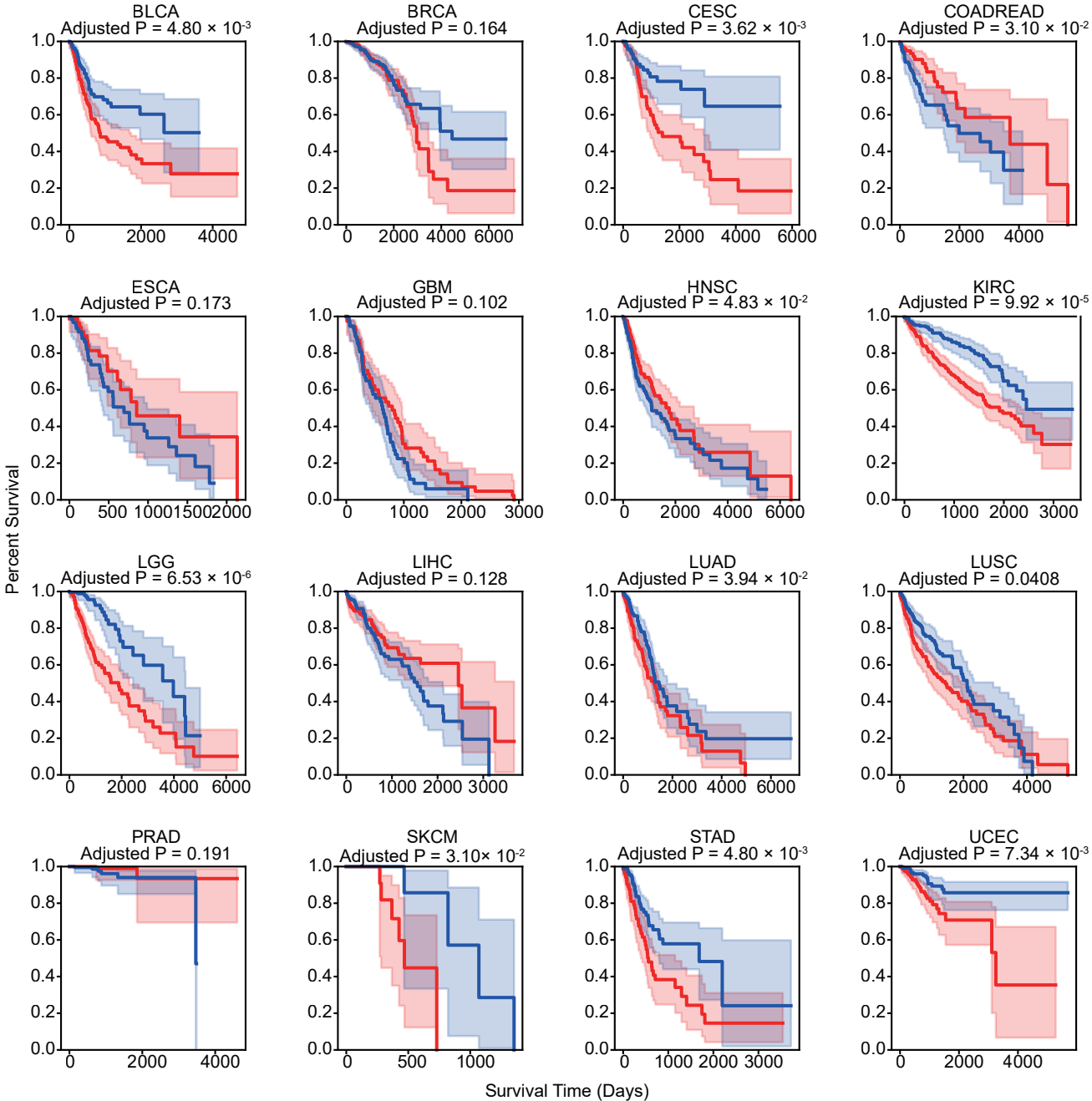

Supplement: qzaf070_Supplementary_Data [file qzaf070_supplementary_data.zip › Figure_S13.pdf]

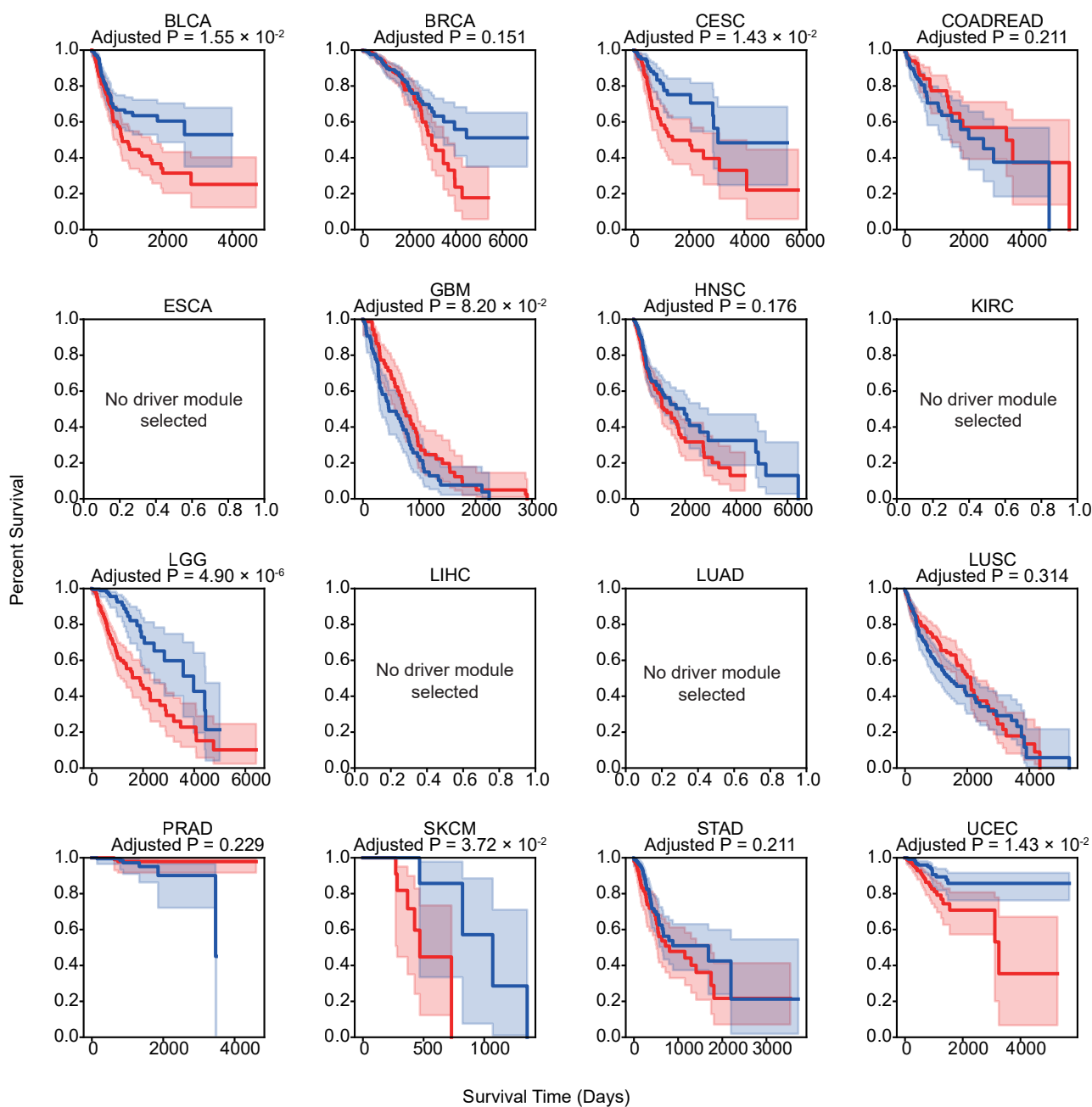

Supplement: qzaf070_Supplementary_Data [file qzaf070_supplementary_data.zip › Figure_S14.pdf]

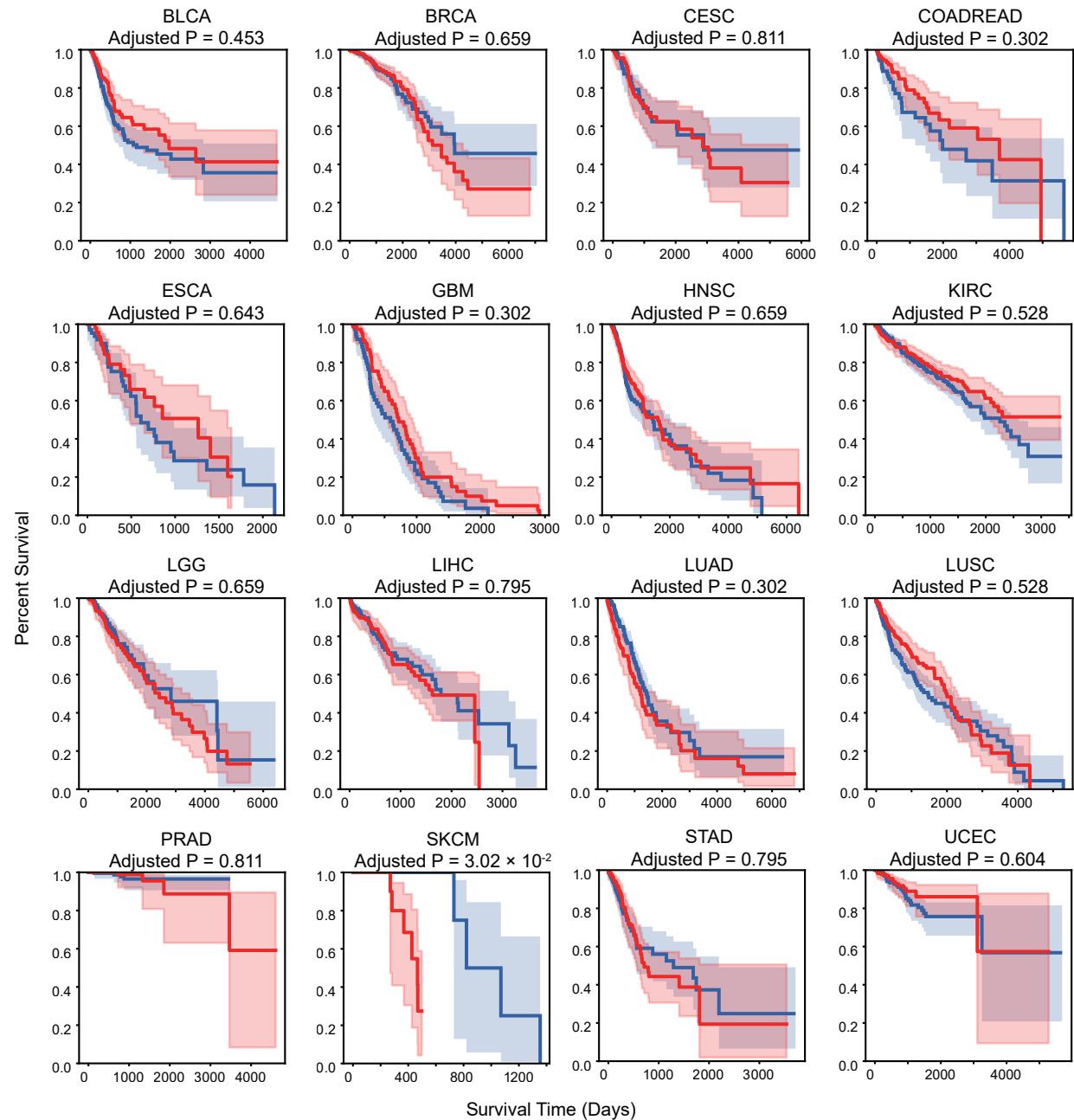

Supplement: qzaf070_Supplementary_Data [file qzaf070_supplementary_data.zip › Figure_S15.pdf]

**A**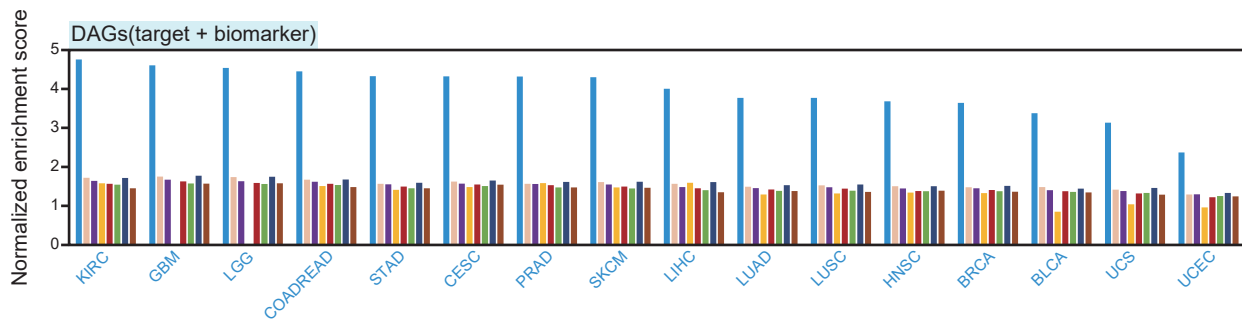**B**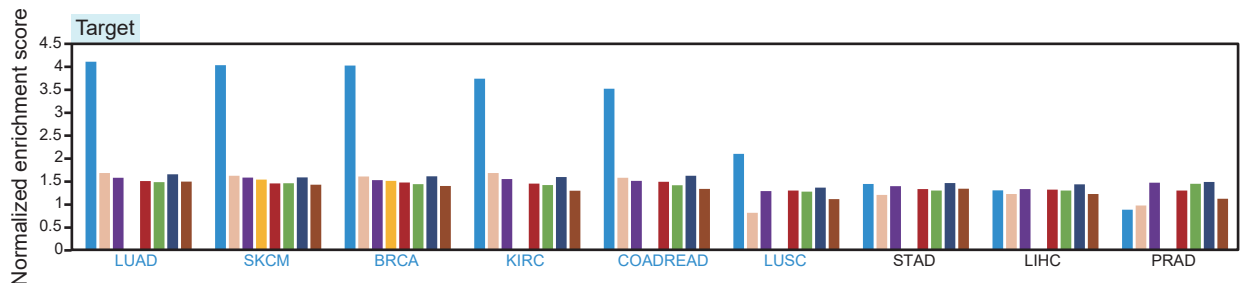**C**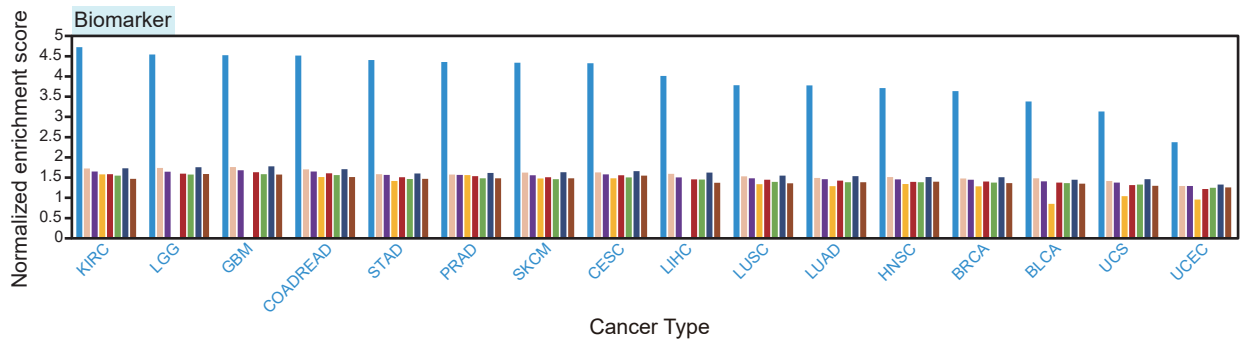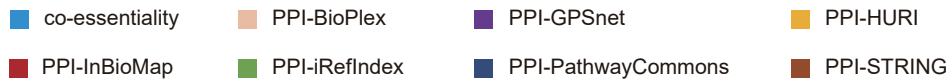

Supplement: qzaf070_Supplementary_Data [file qzaf070_supplementary_data.zip › Figure_S17.pdf]

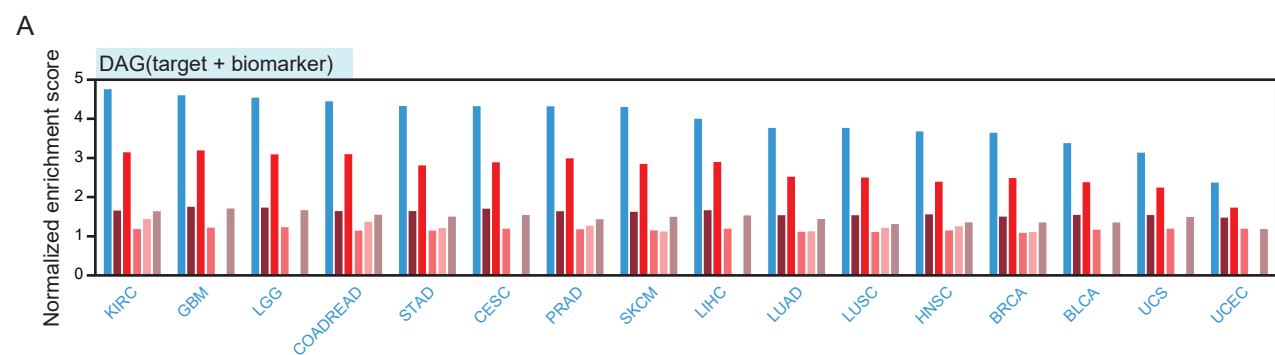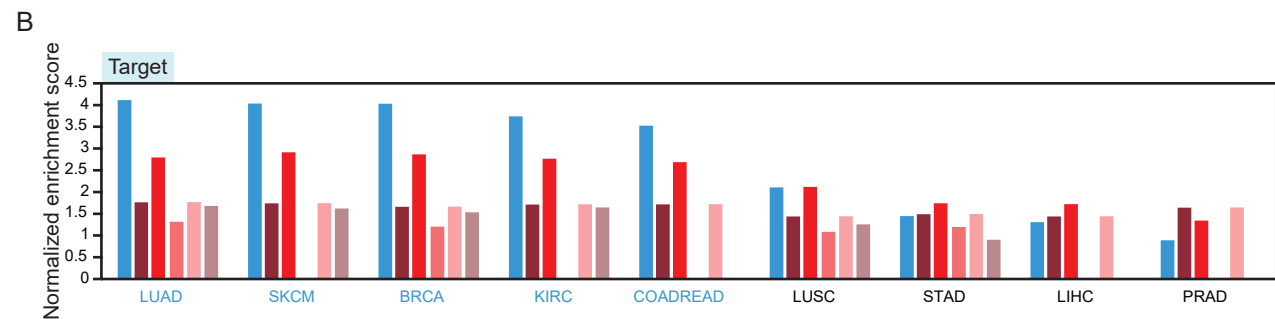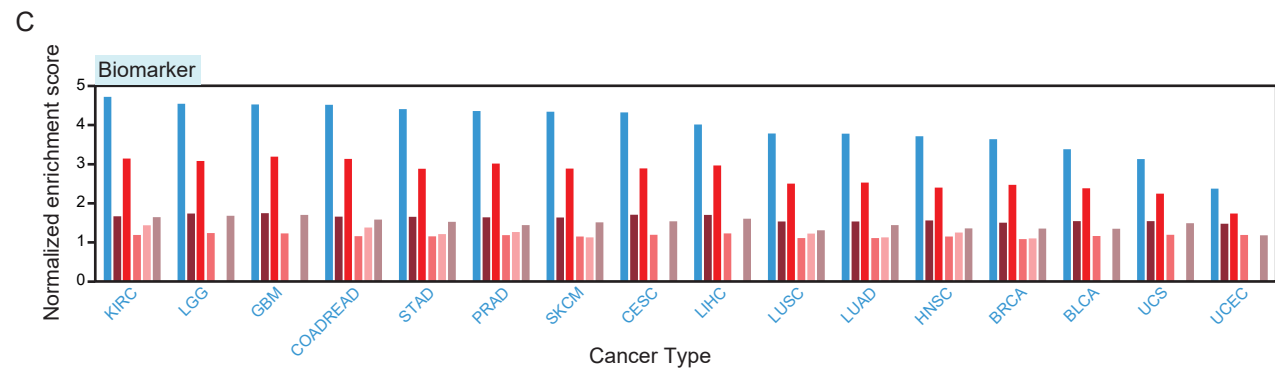

co-essentiality    Wainber\_et al    Amici\_et al  
 Gheorghe\_et al\_Ceres    Gheorghe\_et al\_BF    cSLnet

Supplement: qzaf070_Supplementary_Data [file qzaf070_supplementary_data.zip › Figure_S18.pdf]

A

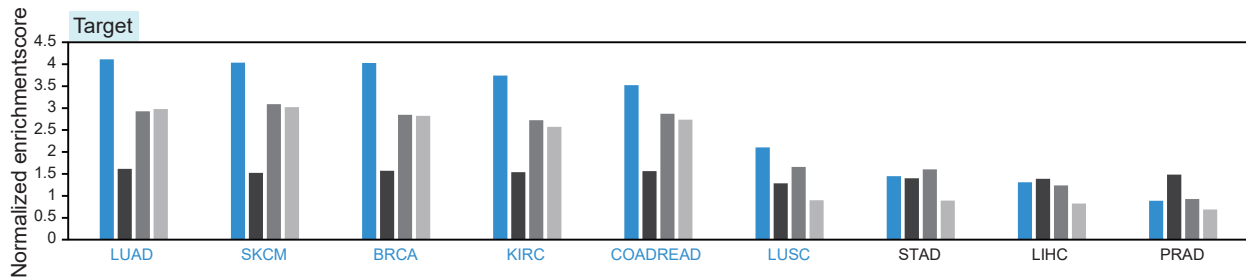

B

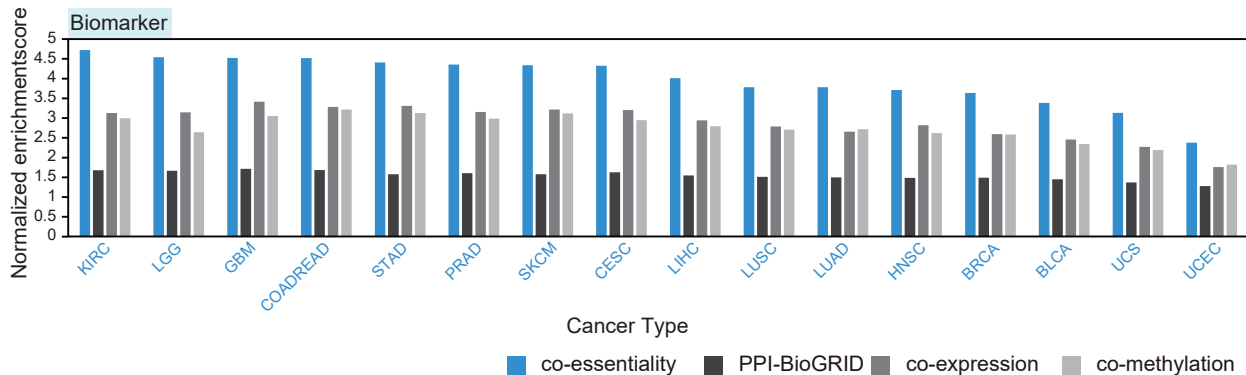

Supplement: qzaf070_Supplementary_Data [file qzaf070_supplementary_data.zip › Figure_S19.pdf]

**A**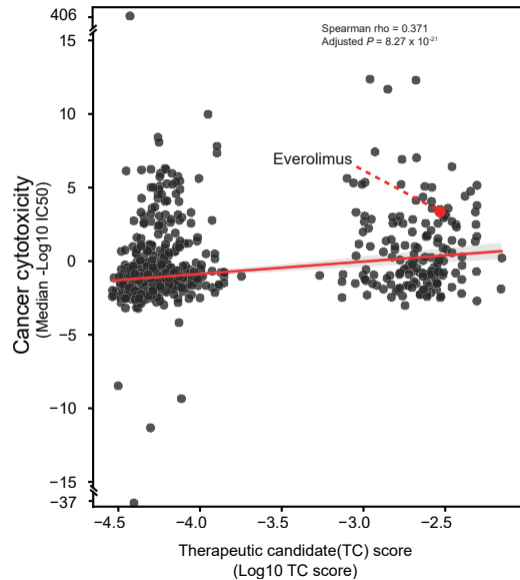**B**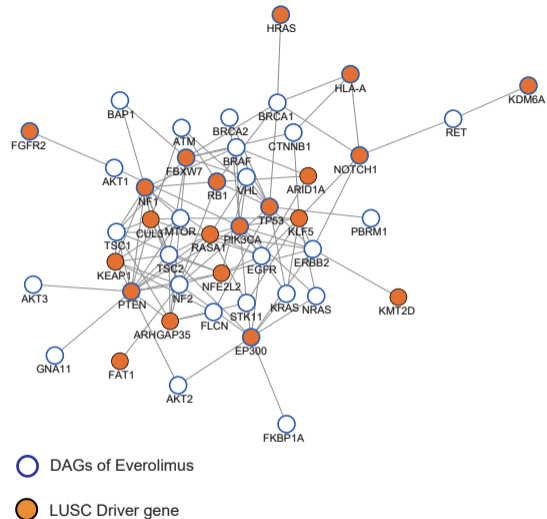

Supplement: qzaf070_Supplementary_Data [file qzaf070_supplementary_data.zip › Figure_S21.pdf]

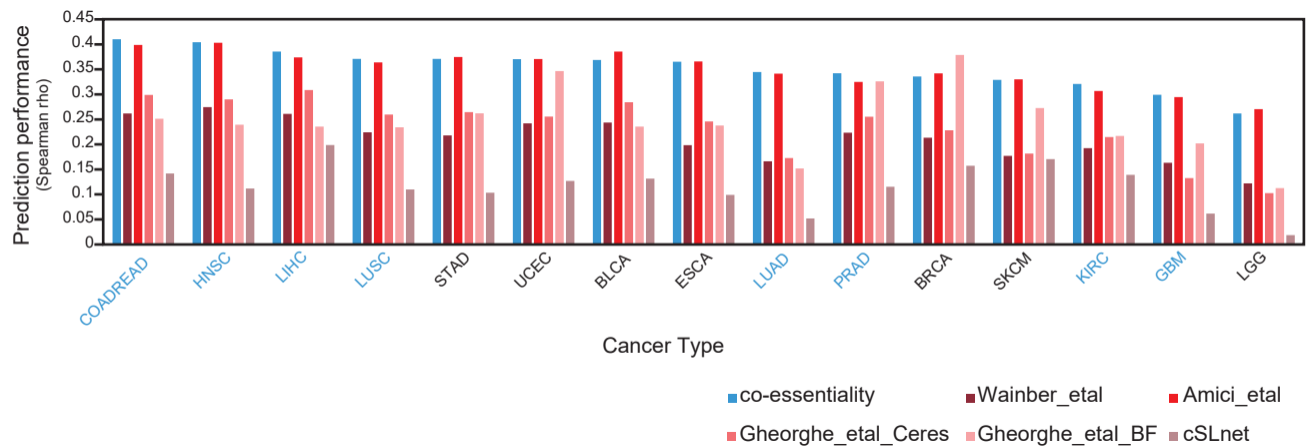

Supplement: qzaf070_Supplementary_Data [file qzaf070_supplementary_data.zip › Figure_S23.pdf]

Prediction performance  
(Spearman rho)

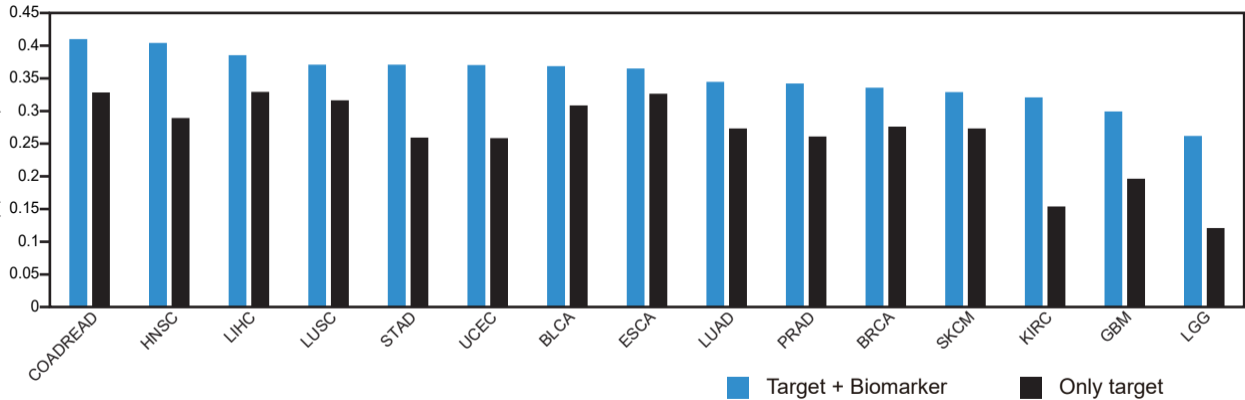

Supplement: qzaf070_Supplementary_Data [file qzaf070_supplementary_data.zip › Figure_S24.pdf]

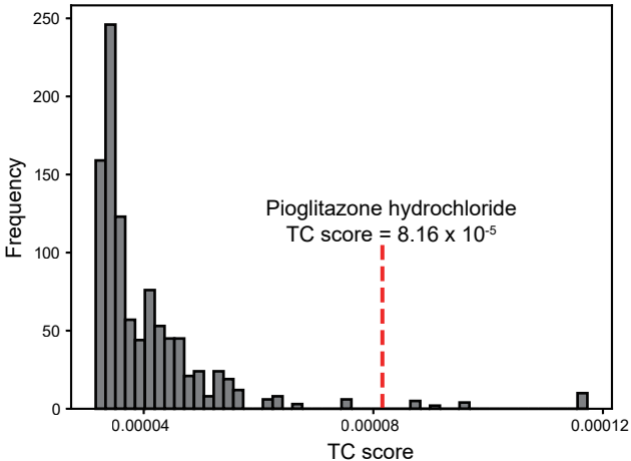

Supplement: qzaf070_Supplementary_Data [file qzaf070_supplementary_data.zip › Figure_S25.pdf]

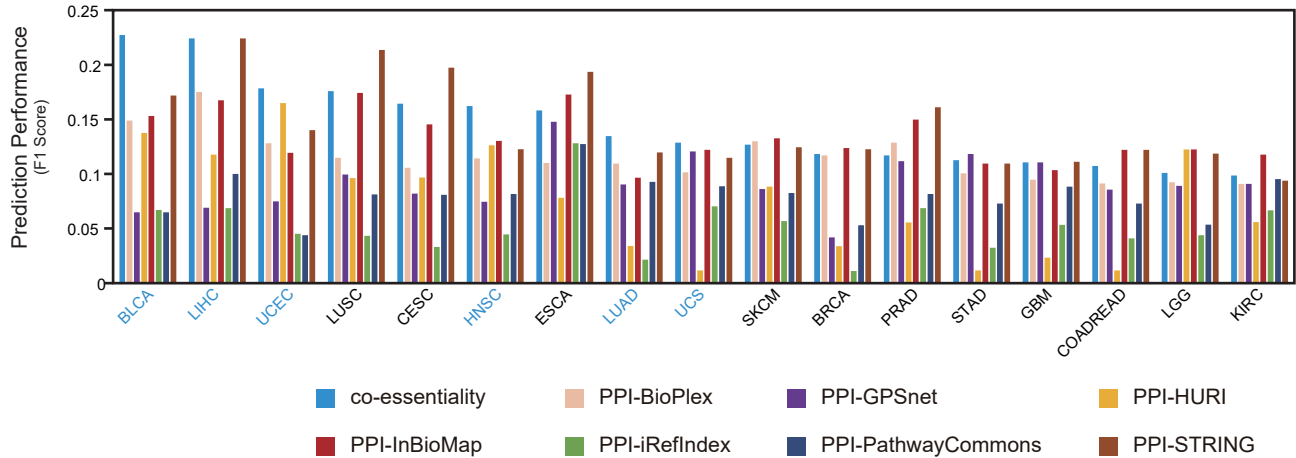

Supplement: qzaf070_Supplementary_Data [file qzaf070_supplementary_data.zip › Figure_S26.pdf]

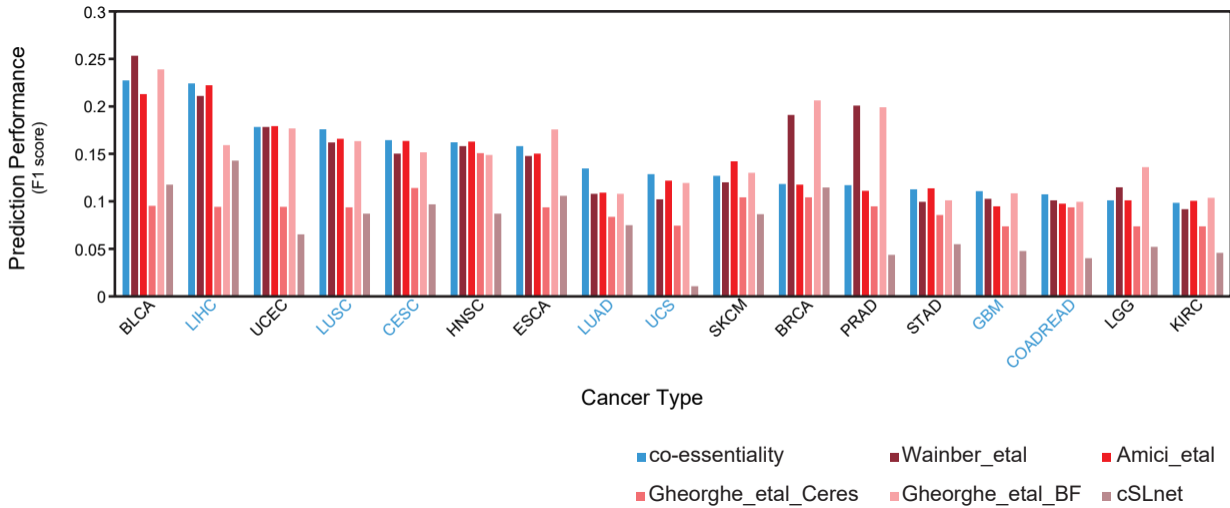

Supplement: qzaf070_Supplementary_Data [file qzaf070_supplementary_data.zip › Figure_S27.pdf]

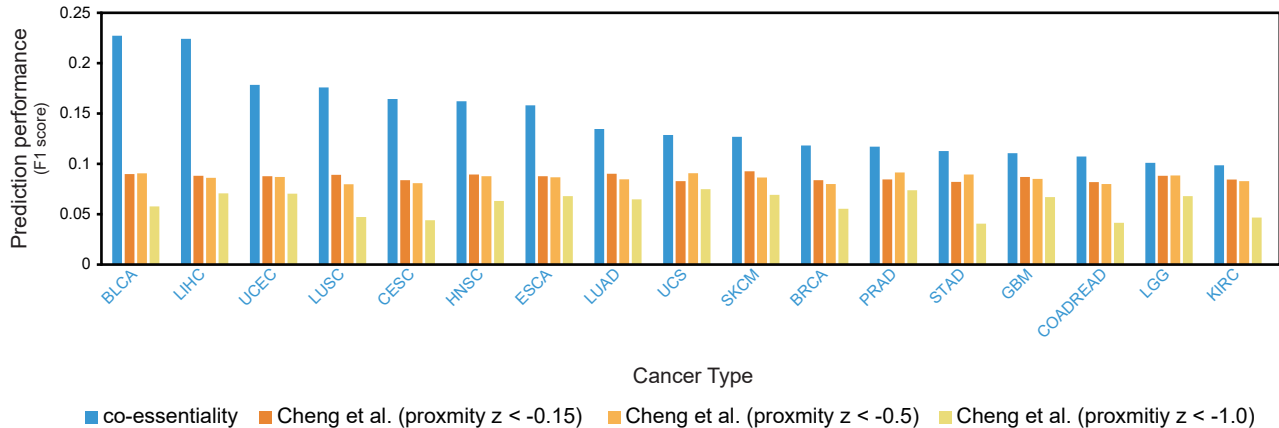

Supplement: qzaf070_Supplementary_Data [file qzaf070_supplementary_data.zip › Figure_S28.pdf]

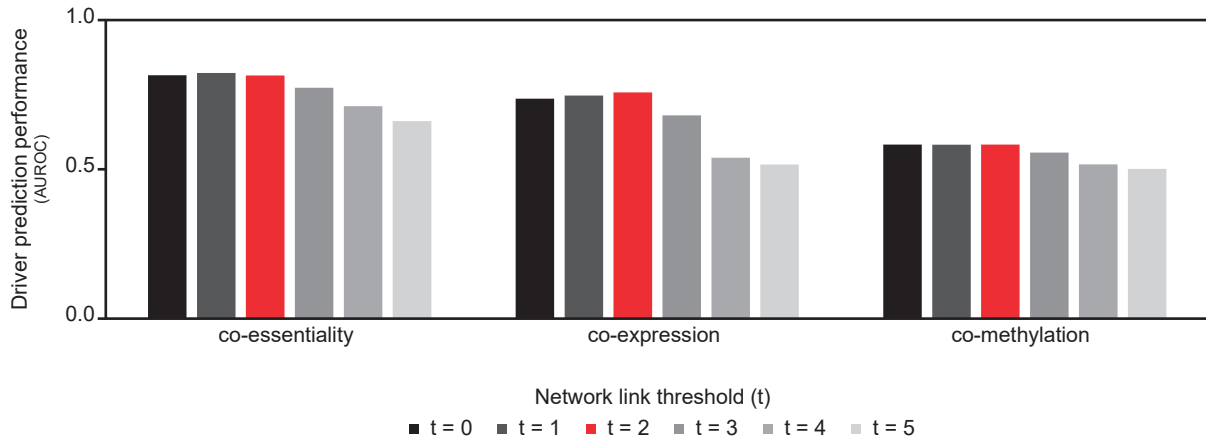

Supplement: qzaf070_Supplementary_Data [file qzaf070_supplementary_data.zip › Figure_S29.pdf]

Link enrichment to functional module

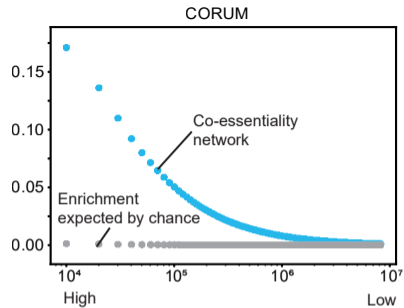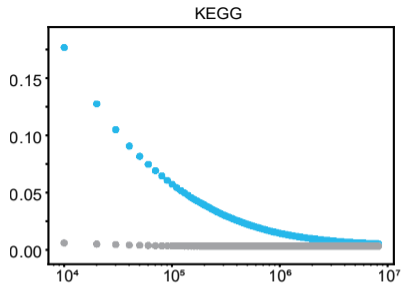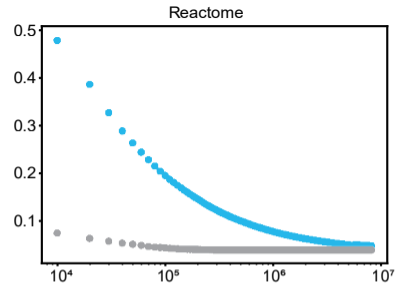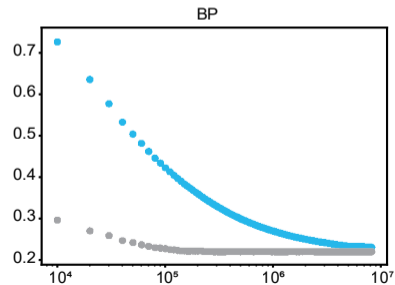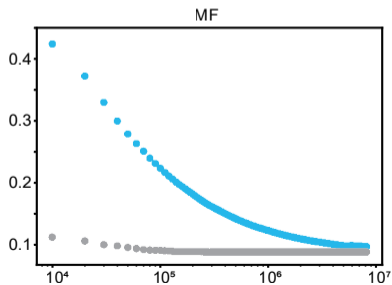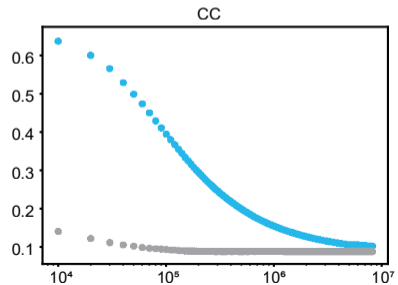

Rank of co-essentiality link weight

Supplement: qzaf070_Supplementary_Data [file qzaf070_supplementary_data.zip › Figure_S1.pdf]

**A**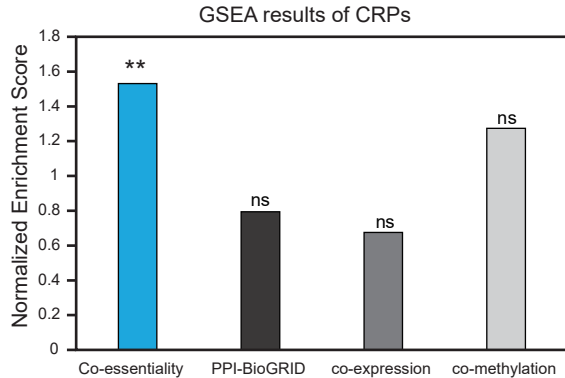**B**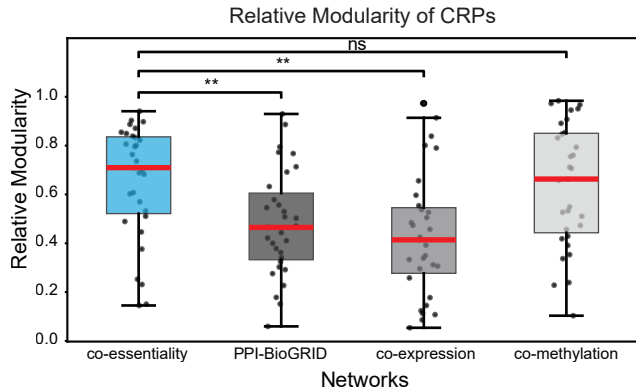

Supplement: qzaf070_Supplementary_Data [file qzaf070_supplementary_data.zip › Figure_S2.pdf]

co-essentiality

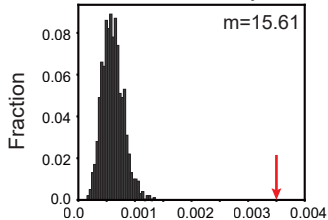

PPI-BIOGRID

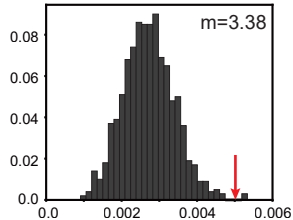

co-expression

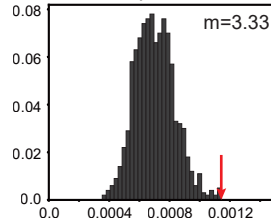

co-methylation

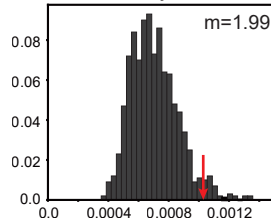

Cohesiveness (modularity)

Supplement: qzaf070_Supplementary_Data [file qzaf070_supplementary_data.zip › Figure_S3.pdf]

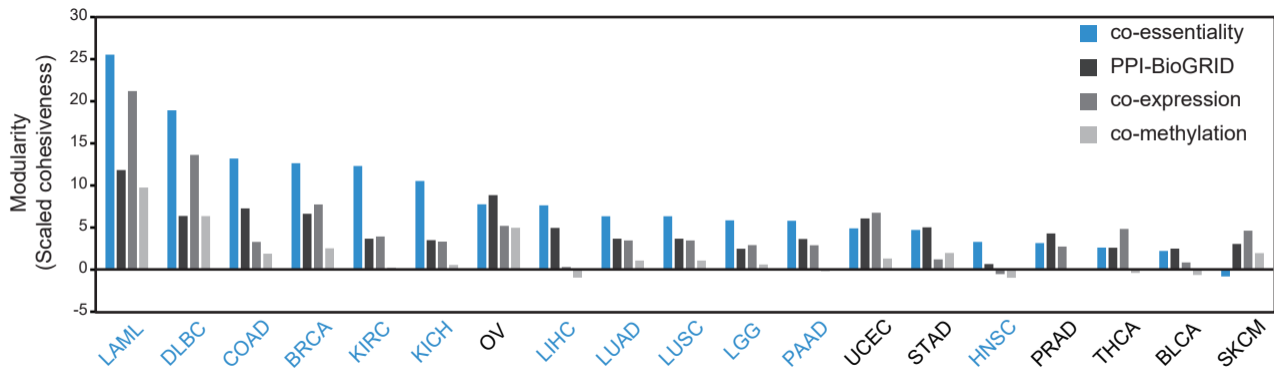

Supplement: qzaf070_Supplementary_Data [file qzaf070_supplementary_data.zip › Figure_S4.pdf]

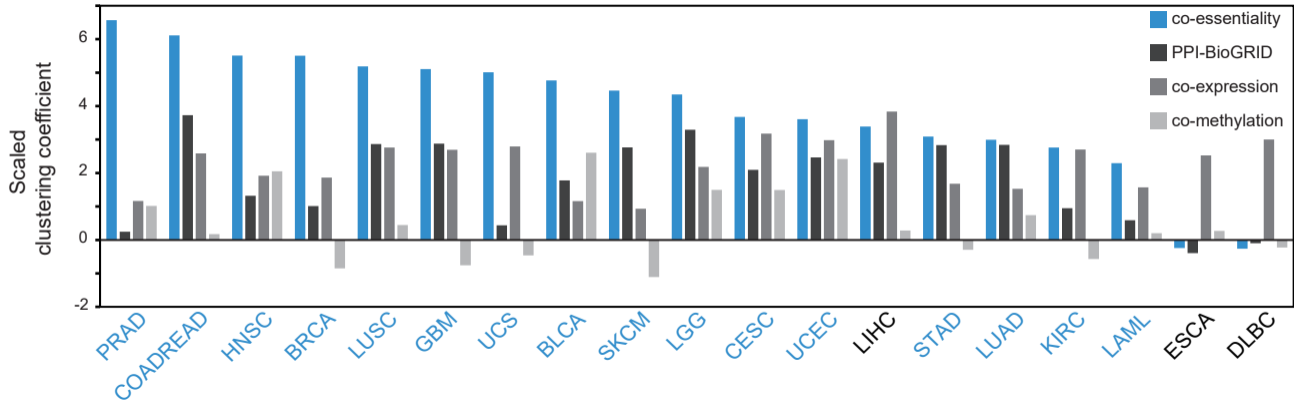

Supplement: qzaf070_Supplementary_Data [file qzaf070_supplementary_data.zip › Figure_S5.pdf]

Modularity  
(Scaled cohesiveness)

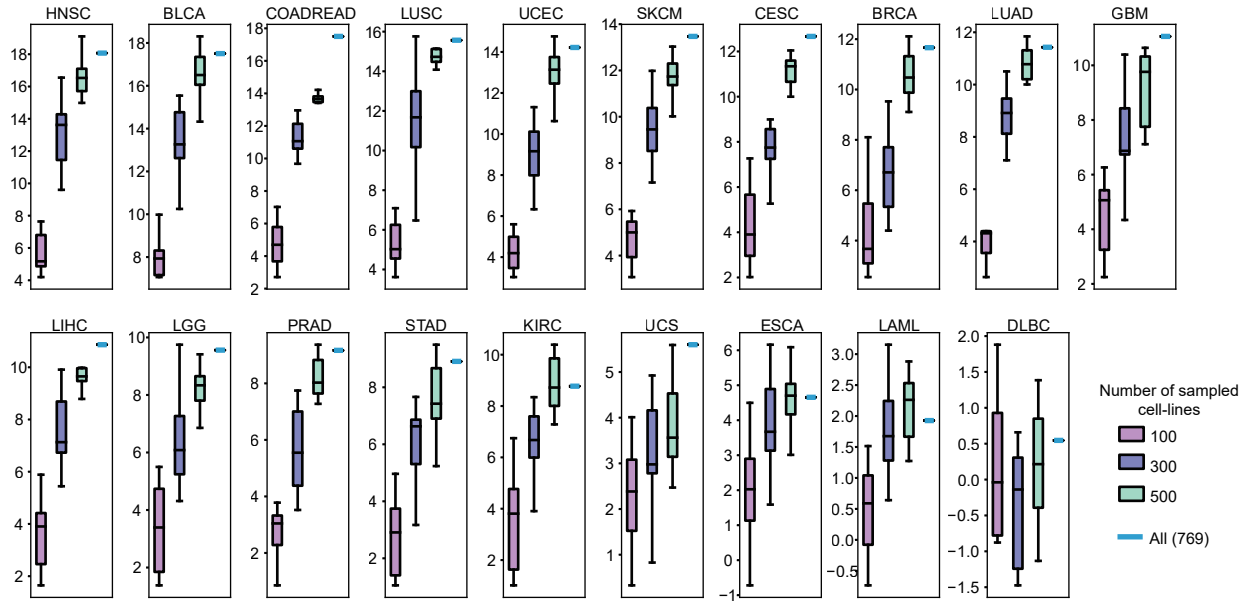

Supplement: qzaf070_Supplementary_Data [file qzaf070_supplementary_data.zip › Figure_S6.pdf]

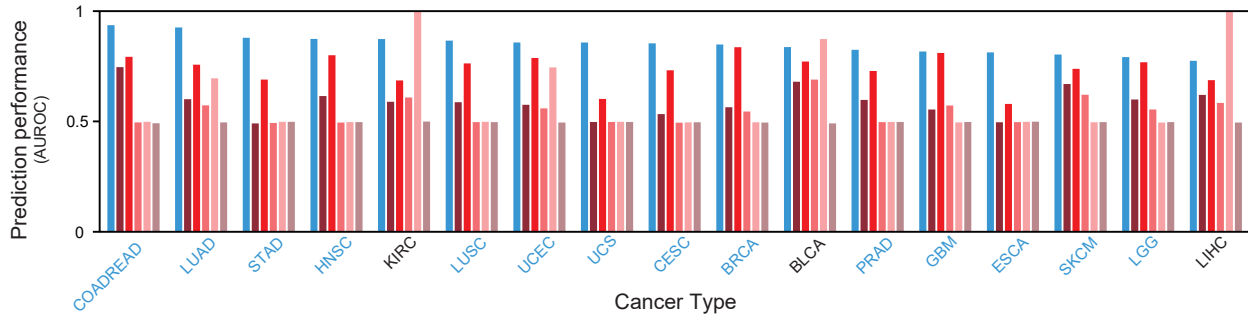

co-essentiality

Wainber\_etal

Amici\_etal

Gheorghe\_etal\_Ceres

Gheorghe\_etal\_BF

cSLnet

Supplement: qzaf070_Supplementary_Data [file qzaf070_supplementary_data.zip › Figure_S8.pdf]
